# Whole Genome or Single Genes? A Phylodynamic and Bibliometric Analysis of PRRSV

**DOI:** 10.3389/fvets.2021.658512

**Published:** 2021-06-24

**Authors:** Alba Frias-De-Diego, Manuel Jara, Brittany M. Pecoraro, Elisa Crisci

**Affiliations:** Department of Population Health and Pathobiology, College of Veterinary Medicine, North Carolina State University, Raleigh, NC, United States

**Keywords:** bibliometrics, phylodynamics, pig, PRRSV, ORF5, whole genome

## Abstract

Diversity, ecology, and evolution of viruses are commonly determined through phylogenetics, an accurate tool for the identification and study of lineages with different pathological characteristics within the same species. In the case of PRRSV, evolutionary research has divided into two main branches based on the use of a specific gene (i.e., ORF5) or whole genome sequences as the input used to produce the phylogeny. In this study, we performed a review on PRRSV phylogenetic literature and characterized the spatiotemporal trends in research of single gene vs. whole genome evolutionary approaches. Finally, using publicly available data, we produced a Bayesian phylodynamic analysis following each research branch and compared the results to determine the pros and cons of each particular approach. This study provides an exploration of the two main phylogenetic research lines applied for PRRSV evolution, as well as an example of the differences found when both methods are applied to the same database. We expect that our results will serve as a guidance for future PRRSV phylogenetic research.

## Introduction

Viral diseases affecting livestock are a major problem because of their rapid spread, negative impact in animal health, potential spillover to humans, and detrimental effect on economic systems (1, 2). In 2019, international commerce of livestock and swine products surpassed $20 billion worldwide, from which the U.S. alone reached over $7 billion as reported by the United States Department of Agriculture (USDA) (3). For those reasons, controlling infectious diseases affecting swine is an ever-growing challenge, shaped by the constant race between the potential of pathogens to evolve and spread, and the ability of researchers to elucidate mechanisms and to develop effective prophylactic and therapeutic measures to reduce their impact on the swine population.

Viruses in general are one of the infectious agents with the highest mutation rates, which hinders the ability of researchers to predict their evolution and spread due to their ever-changing genome (4, 5). This is particularly common in the case of RNA viruses such as Porcine Reproductive and Respiratory Syndrome Virus (PRRSV), currently one of the most deleterious diseases for the swine industry worldwide, reaching up to 60% prevalence in growing-finishing herds (1, 2, 6, 7). PRRSV is an enveloped positive sense single-stranded RNA virus in the family Arteriviridae (8) with a genome of ~15 kb that encodes at least nine open reading frames (ORFs) (9). ORF5 in particular is widely used for phylogenetic analysis, since its structure encompasses both hypervariable and conserved segments, allowing the classification of strains in a reasonably accurate way (10–12). Based on its genetic diversity and antigenic properties, PRRSV is classified into two distinct genotypes with different species (13): Type 1 and Type 2, that are mostly circulating in Europe and North America respectively (11, 14). Due to its genetic nature, its recombination ability is one of the main shapers of PRRSV evolution and diversity (15, 16), along with its mutation rate, that was recently estimated at 0.00672 (16).

Over the last decade, the concept and application of interdisciplinary sciences have improved infectious disease control measures by the combination of the genetic, geographic, and historical data of pathogens (17–20), providing a much deeper understanding of their evolutionary trajectory and therefore allowing the application of targeted control strategies and treatments based on this new information (16). However, due to the multiple possibilities that interdisciplinary approaches offer, there is often an open discussion to determine the most accurate method to apply in a given scientific scenario.

Early molecular studies of PRRSV applied RT-PCR to detect virus, leading to the first PRRSV phylogenetic reconstruction based on ORF-5 and ORF-7 sequences that was able to differentiate the European and American clades (21). This paper was followed by numerous phylogenetic studies using ORF-5 given the compromise of sites evolving at different rates, which generated well-resolved trees during a period where whole genome sequencing was particularly challenging. However, despite the increasing availability of whole genome sequences, scientists are still divided by the support of the use of whole genomes or single genes (22), particularly for evolutionary analyses of organisms like PRRSV, that is an exceptionally diverse virus (23).

Multiple factors have a key role in this decision, especially for research analyzing field isolates and samples that need to be sequenced, since the economic effort needed, along with the requirement of specialized laboratories, equipment and skilled personnel is remarkably higher to perform whole genome sequencing than single genes (24). In the case of whole genome defenders, they advocate the consideration of all nucleotides to identify all changes between genomes, rather than the changes in specific and usually conserved genes (caused for example by horizontal gene transfer) (25, 26). On the other hand, opinions of scientists supporting the use of single genes or multi-gene approaches (but not whole genome application) (27, 28) argue that by considering whole genomes, there is the possibility to detect changes in non-coding genes that could misclassify sequences.

The goal of this study is to evaluate and compare the different patterns and trends on PRRSV research in relation to the application of whole genomes or single genes, and assess the potential variations observed on the same analyses when one of the two approaches is applied using the same genetic database.

## Materials and Methods

### Bibliometric Analyses

The bibliometric search for the available publications was performed in Scopus, using the search criteria “TITLE-ABS-KEY [(PRRSV AND [whole AND genome OR ORF5]) OR (porcine AND reproductive AND respiratory AND syndrome AND [whole AND genome OR ORF5]) OR (PRRSV AND [phylogeny OR phylogenetics OR evolution])]” and downloaded the obtained results in bib format. Using the R package Bibliometrix (29), the journal, year of publication, title, abstract, author names, and author affiliations of all resulting publications were extracted. From this initial database, a manual screening was performed: original or literature reviews, the study area (global vs. country level), and the use of whole genome or ORF5 gene were extracted.

### Genetic Databases

All PRRSV whole genome sequences available were downloaded from Genbank as a.gb file and ran the python package “gbmunge” (https://github.com/sdwfrost/gbmunge) to retrieve the available metadata for each sequence. From that database, 765 sequences, for which geographic and temporal information was available, were selected for subsequent analyses. Sequences were aligned using MEGA X (30). Using this updated database, the sequences were aligned along with three PRRSV ORF5 sequences, EU556220, DQ405282, and DQ405279, to identify the region of the whole genome in which ORF5 was located. Then, ORF5 region of the sequences was manually identified and saved in a second database used for analyses.

### Recombination Analyses

The recombination detection program (RDP) v5.3 was used to search for recombination within our data set (31). The alignment was screened using five methods (BootScan, Chimaera, MaxChi, RDP, and SiScan).

### Phylogeny

To find the best substitution model for each database, the ModelFinder tool (32) built into IQ-Tree version 1.6.1 (33) was used. The marginal likelihood value supported the use of the general time-reversible model (GTR) with gamma-distributed rate heterogeneity plus a proportion of invariable sites (GTR+G+I) (34) for both databases (Supplementary Table 1). To determine the best fitting node-age and branch-rate model, each combination of molecular clock and branch rate was run to compare the marginal likelihood estimated by the stepping-stone and path-sampling methods, supporting the use of uncorrelated relaxed molecular clock model and coalescent logistic growth as the tree prior for both ORF-5 and whole genome databases. Both phylogenies were then estimated by Bayesian inference through Markov chain Monte Carlo (MCMC), implemented in BEAST v2.6.0 (35). The model was run for 100 million generations, sampling every 10,000th generation and removing 10% of the chain as burn-in in both cases. The probabilities of ancestral states were inferred from the Bayesian discrete-trait analysis and visualized as pie charts on each node. Visualization of the trees was performed using FigTree v1.4.4 (Rambaut^1^).

### Phylodynamics

The spatiotemporal spread patterns observed for both databases were performed via Bayesian continuous phylogeographic analysis, following the model selection described in the phylogeny section. An uncorrelated relaxed molecular clock model with lognormal distribution (36) and the Bayesian SkyGrid with covariates as the coalescent tree prior (37) were also applied. To ensure an effective sample size (ESS) value over 200, analyses were run for 200 million generations, sampling every 10,000th generation and removing 10% of the chain as burn-in. To determine the relative genetic diversity over time of each database we used Bayesian SkyGrid, as this approach relies on a non-parametric coalescent model to estimate the effective population size over time (38).

## Results

### Bibliometric Analyses and Genetic Databases

The bibliometric search recognized 374 articles under our search criteria, from which only 49 were global studies. From that total, 23 literature reviews and 351 original articles, from which 190 of them applied whole genome analyses, and 155 used single genes, were identified. Detailed information about the 6 remaining articles was not available at the time of the screening (October 2020). One hundred and twenty five of the publications using single genes chose ORF5, leaving only 30 articles with a different genome section [i.e., ORF7 (10), Nsp2 (39)].

**Figure 1:**
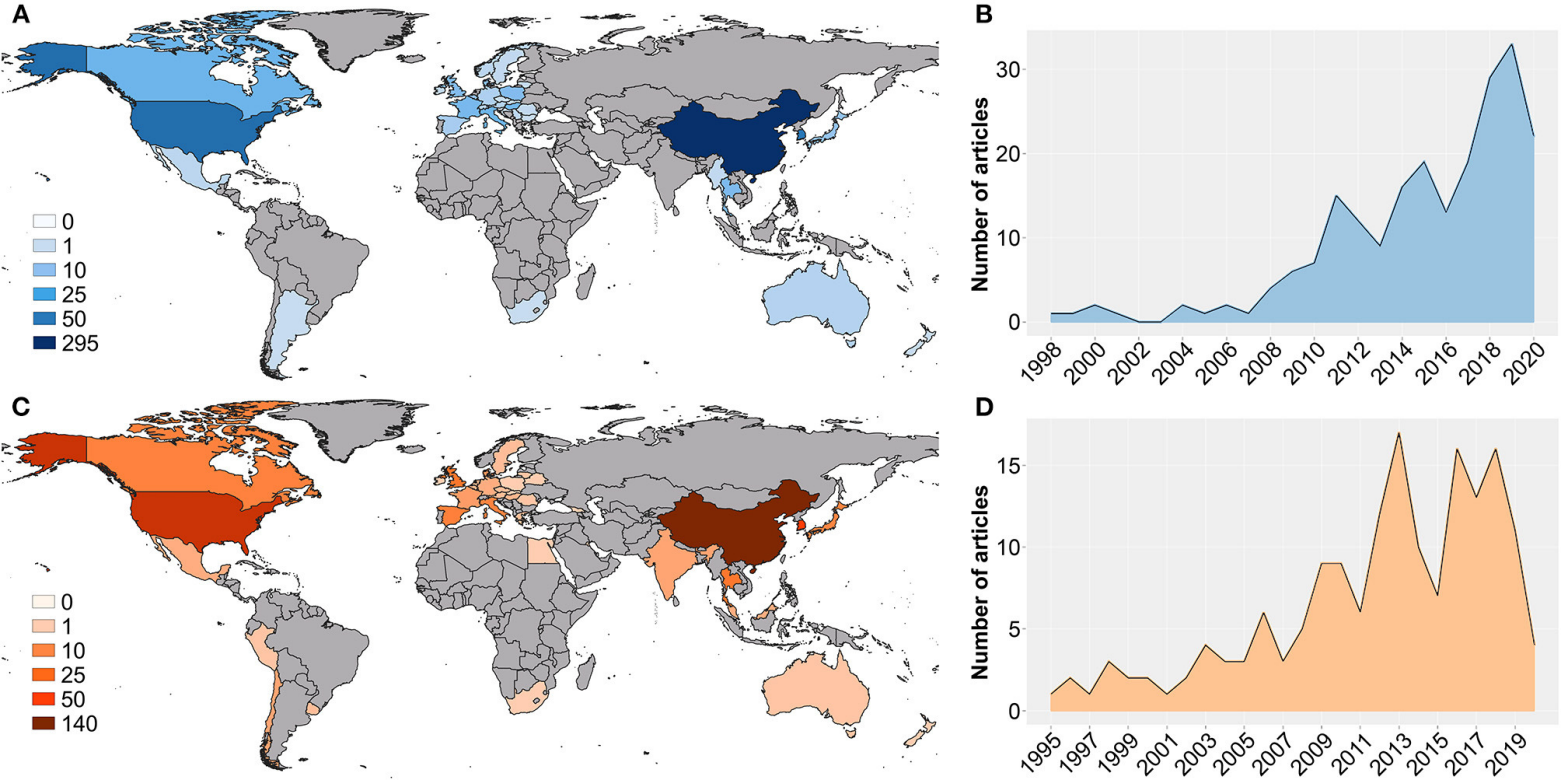
Global annual scientific production involving whole genome sequences (blue) and single genes (orange) where **A** represents the number of publications per country of whole genome-related research, **B** represents the global annual scientific production of whole genome-related research, **C** represents the number of publications per country of single genes-related research, and **D** represents the global annual scientific production of single genes-related research.

For both whole genome sequences (WGS) and ORF5 sequences, the countries with the highest scientific productivity were China (WGS = 295, ORF5 = 141), the United States (WGS = 89, ORF5 = 88), and South Korea (WGS = 55, ORF5 = 50) (Figure 1A). Overall, PRRSV research (whole and partial genome studies) evidenced higher scientific productivity per year up to 2018, with a rapid decrease maintained until our search was performed (October 2020) (Figure 1B, Supplementary Figure 1). In addition, 12 countries only evidenced articles using WGS, while 8 countries only produced articles related to ORF5 sequences (Supplementary Table 2).

In relation to the produced genetic databases, the starting point after running the gbmunge package was of 765 whole genome PRRSV sequences with most of the necessary metadata available (Supplementary Table 1). To avoid sampling bias, a similar number of sequences was chosen from different locations to reach a total of 100. The ORF5 database consisted of the exact same sequences from which the ORF5 section of the genome was manually identified and isolated.

### Recombination Analyses

From the total number of sequences with metadata we obtained (765), the Recombination Detection Program (RDP) detected 491 recombinant sequences from the whole genome database, and 393 from the ORF5 database (Figure 1), making a difference of 98 between the two (data available upon request).

### Phylogeny and Ancestral Reconstruction

**Figure 2:**
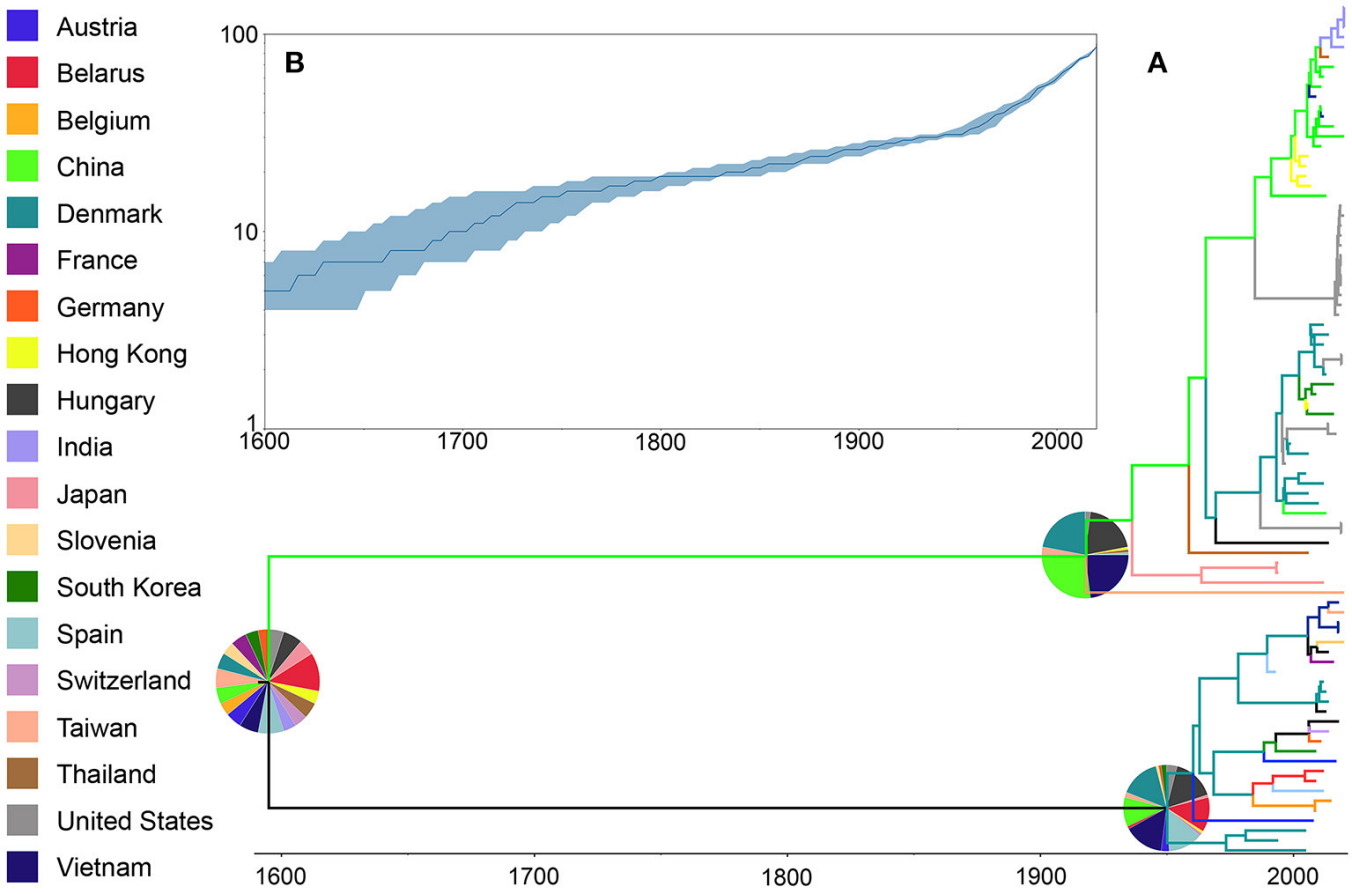
Phylogenetic history of PRRSV inferred using whole genome sequences. **(A)** Maximum clade credibility (MCC) phylogeny, colored according to the country of origin of each sequence. The probabilities of ancestral states (inferred from the Bayesian discrete-trait analysis) are shown in pie charts on each node. **(B)** Spatiotemporal patterns represented through lineages through time plot.

Phylogenetic results for the whole genome database showed that the most likely center of origin for the PRRSV sequences analyzed was Belarus with a root state posterior probability (RSPP) = 10%. This original lineage then diverged into two groups likely driven by geographical distance and independent subsequent evolution, one with a higher probability of being originated in China (RSPP = 26%), and a second one likely originated in Hungary (RSPP = 16%) (Figure 2A). The amount of lineages present over time showed an overall increase in the number of different lineages with two main periods of growth from 1600 to 1750 and from 1950 to the present day (Figure 2B).

**Figure 3:**
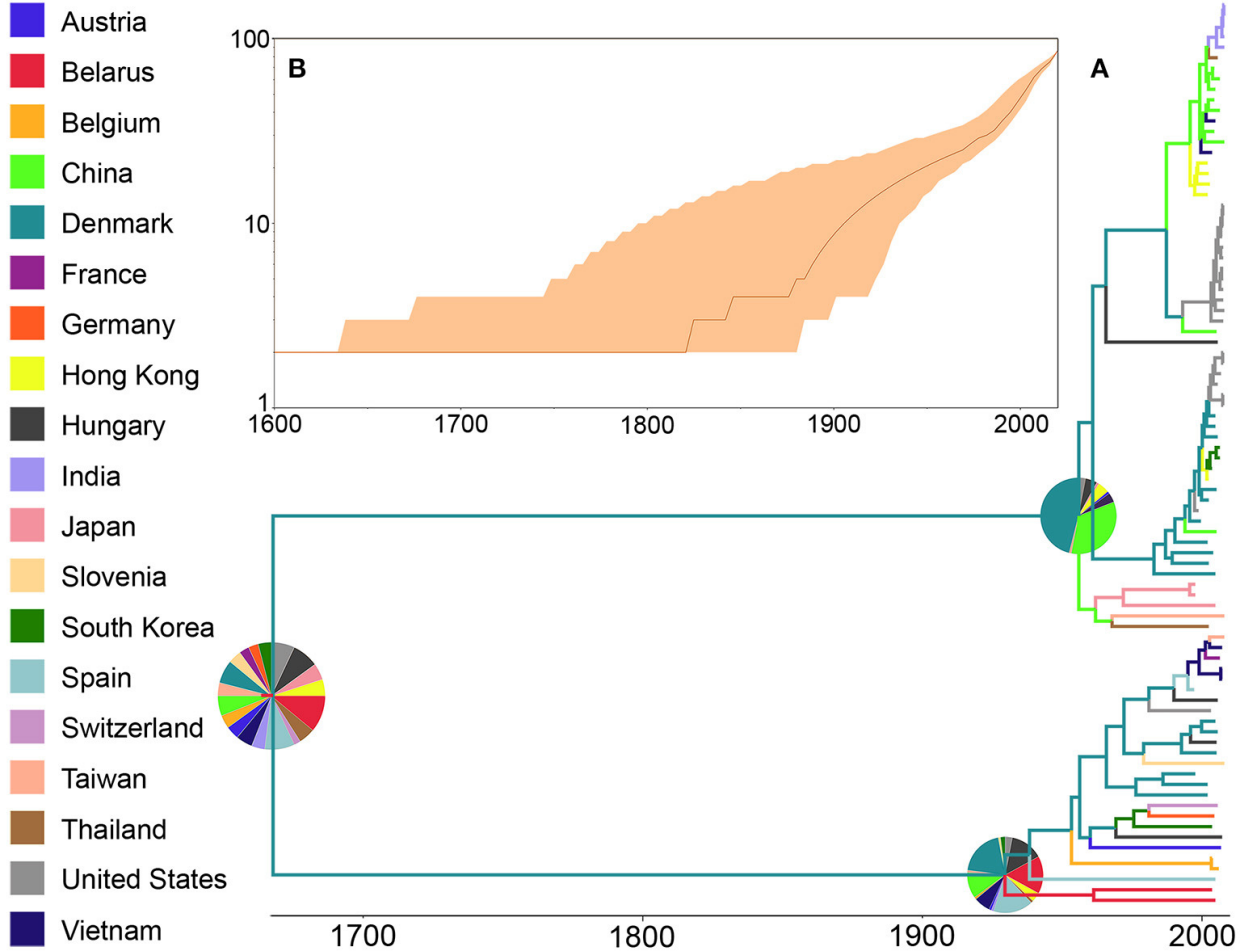
Phylogenetic history of PRRSV inferred using ORF5 sequences. **(A)** Maximum clade credibility (MCC) phylogeny, colored according to the country of origin of each sequence. The probabilities of ancestral states (inferred from the Bayesian discrete-trait analysis) are shown in pie charts on each node. **(B)** Spatiotemporal patterns represented through lineages through time plot.

When the ORF5 sequence database was analyzed, the ancestral reconstruction also evidenced Belarus as the most likely country of origin (RSPP = 11%) (Figure 3). Similarly to the pattern shown by WGS, the analysis showed that this ancestral lineage diverged into two clearly defined groups, both of them with a higher probability of being originated in Denmark (RSPP = 45, and 26% respectively). In the case of the number of lineages through time, this database showed no new lineages appearing until after 1,800 when it presented two clear isolated diversification events that maintained the number of lineages until ~1,900, which was the starting point of a rapid exponential increase in the number of lineages up to the day of the screening (Figure 3B). Finally, the 95% highest probability density (HPD) values of both trees showed similar uncertainty patterns, with the most recent nodes showing less uncertainty than the ancestral (Supplementary Figure 2). However, based on these HPD values the ORF-5 database showed less accuracy to reconstruct the ancestral nodes, suggesting whole genome as the most robust approach for this type of studies.

### Phylodynamics

**Figure 4:**
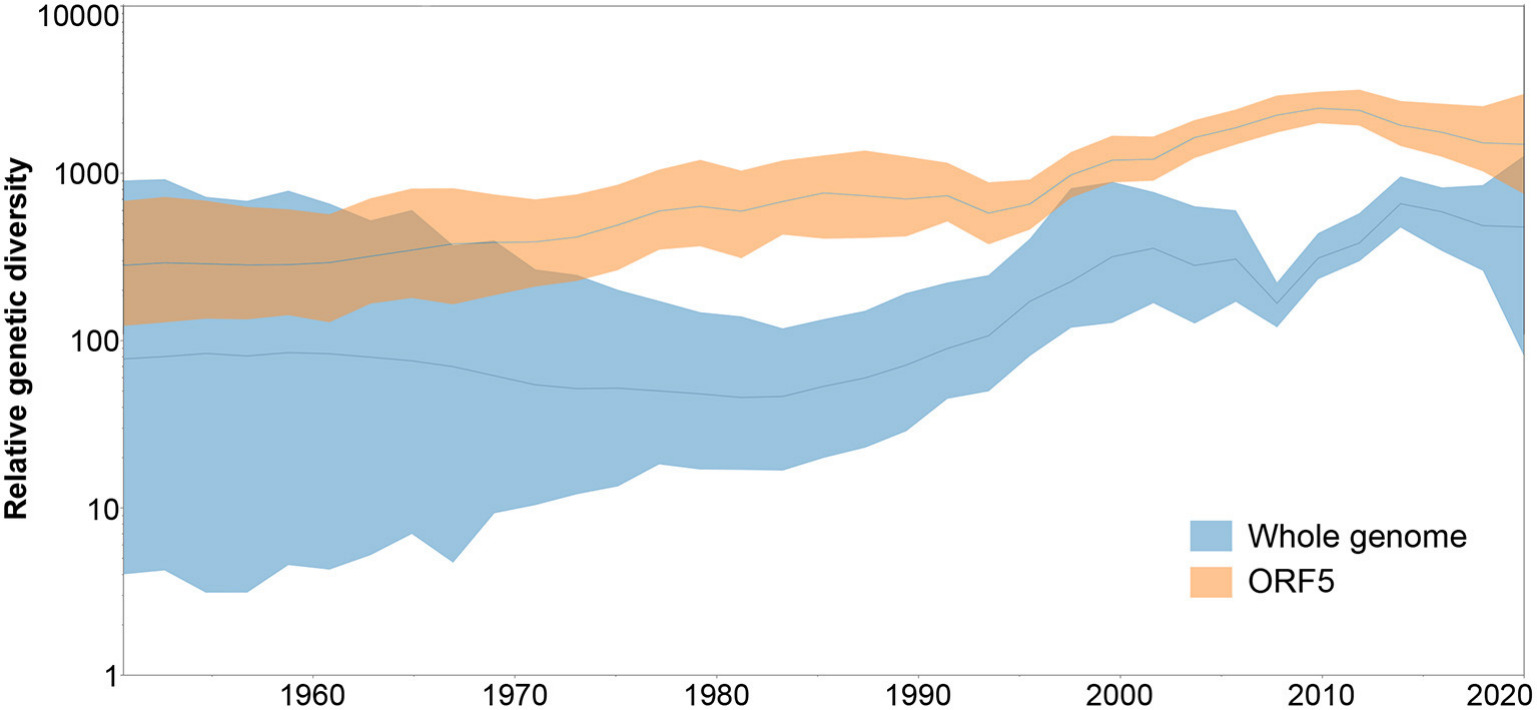
Spatiotemporal patterns in the relative genetic diversity represented through the Bayesian SkyGrid plot, where dark lines represent the mean values, while shaded light regions correspond to the 95% highest posterior density (HPD).

When the genetic diversity obtained for the analysis of whole genome vs. ORF5 databases was compared over time, SkyGrid plot revealed an overall higher genetic diversity exhibited by the ORF5 sequences. In addition, there is a noticeable difference in the pattern of diversity increase, where the ORF5 database showed constant growth while the whole genome database started to experience an increase in diversity from 1983, with a sharp decrease in 2009 that was not detected by the ORF5 result (Figure 4).

Finally, when we compared the dispersal velocity of PRRSV based on each database, we observed that ORF5 sequences showed higher spread velocity (2948.7 km/year) than whole genome sequences (1956.4 km/year) (Supplementary Table 3).

## Discussion

Our bibliometric screening detected an overall higher number of articles considering the use of whole genomes than single genes. However, this happened only after 2010, when the increase in the amount of whole genome related publications started to grow and surpassed the application of ORF5 that had been applied in the previous years. The decrease in productivity observed around 2018 could be the result of the detection of an outbreak of African Swine Fever in China (40), which would likely trigger a deviation on research efforts toward that disease. This growth in whole genome sequence application could have been triggered by the increase in the availability and affordability of RNA-seq technology once it became more accessible for general research, surpassing the levels of the use of ORF5 sequences, that had been posed as the standard gene applied to study PRRSV evolution due to their high variability (11). This hypothesis would also fit with our observation on country productivity, where countries with access to sequencing are leading scientific production for both whole genome studies and single genes. Not surprisingly, these increased publication rates are linked to wealthy economies with high pig production given the elevated cost of sequencing studies, although not every country presented publications in both areas.

The bibliographic search was performed using the Scopus database, as it is a large, multidisciplinary database that includes MEDLINE and has been described to combine the characteristics of both Web of Science and PubMed, allowing an improved service for educational and academic needs, also favoring Natural Science and Biomedical research literature (41). Furthermore, previous research compared the amount of publications retrieved by those three different databases, obtaining a more extensive number of detected publications using Scopus (42).

Another expected result was the number of recombinant sequences detected on each database. Recombinant sequences should be considered when choosing between whole genomes or single genes, particularly in RNA viruses such as PRRSV (12, 43, 44). A common claim between scientists supporting the use of single genes for evolutionary analysis relies on the presence of numerous non-coding regions (introns) that could interfere with those analyses and cause bias on the results. However, we observed that the whole genome database detected a higher number of recombinants. Although there are no studies assessing the proportion of recombinant sequences detected due to non-coding regions, numerous publications have mentioned the implications and importance of considering these non-coding sequences on recombination, evolution, and chromosomal stability assessments (45–47).

The shape of the phylogenetic trees obtained for both databases was similar. This suggests that for analyses focusing on the evolutionary patterns as well as the identification and taxonomy of this virus, both approaches could fulfill the needs of the study. However, in the case of the ancestral reconstruction studies, as well as in the reconstruction of phylodynamic patterns, we observed numerous differences between the two datasets, showing that the choice between whole genome or single genes should be considered carefully depending on the study performed. Particularly in the case of ancestral reconstruction studies, where our whole genome database showed more accuracy on the estimated ancestral nodes (measured as HPD values) than our ORF-5 database. It is important to keep in mind that the main goal of this project was not to perform a phylodynamic study of PRRSV or to determine its ancestral origin, but to identify the similarities and differences observed when identical evolutionary analyses were performed in the same sequences of our database using the whole genome or only its ORF5 segment. Interestingly, with the set of sequences used in our analyses, the evolutionary trends and shape of the trees obtained coincide with previously published studies in PRRSV evolutionary history (11, 16), suggesting that even though higher number of sequences will generally produce more robust analyses, inferences and patterns can be identified with reduced amounts of data as a baseline for subsequent and more elaborated studies.

We faced some limitations during the development of this study. Firstly, the affiliation information extracted by the Bibliometrix R package did not necessarily correspond to the country where the initial research was developed. This is a common limitation in bibliometric studies that has been previously assessed via sensitivity analyses with no significant changes on the obtained results (48). In addition, publications using whole genome sequencing are generally complex and include wide collaboration networks where authors from different locations share resources and data. Therefore, one single article may be detected by bibliometric measurements in more than one country. Likewise, our search only included papers that were already published and available by October 2020. Therefore, the productivity of this year must not be considered final, because papers submitted but not yet published before our search day are likely to get published after our search was done or even in 2021.

Finally, this study represents a comparison of the most commonly applied evolutionary analyses in PRRSV research using whole genome or single gene sequences as an input. Here, we show the similarities and differences on the results driven by the use of the whole genome or the ORF-5 section of the same set of genes, analyze the evolution and patterns of research on each area over time and highlight the need to take these differences into consideration when deciding the most appropriate approach to apply depending on the specific aim of the research performed, particularly in analyses that involve ancestral reconstruction.

## Data Availability Statement

The original contributions generated for the study are included in the article/Supplementary Material, further inquiries can be directed to the corresponding author.

## Author Contributions

EC and AF-D-D were responsible for the conception of the study and manuscript writing and revisions. AF-D-D was responsible for the acquisition of data and data analysis. AF-D-D and MJ were responsible for data analysis and interpretation, manuscript editing, and revisions. BP was responsible for manual data screening and revision of the manuscript. EC was responsible for project supervision and administration. All authors have read and agreed to the published version of the manuscript.

## Conflict of Interest

The authors declare that the research was conducted in the absence of any commercial or financial relationships that could be construed as a potential conflict of interest.

## Footnotes

^1^Rambaut, A. “Figtree v1.4.4. Available online at: http://tree.bio.ed.ac.uk/software/figtree/”.
