# Supplementary material for: Whole Genome or Single Genes? A Phylodynamic and Bibliometric Analysis of PRRSV
Source: Front Vet Sci. 2021 Jun 24;8:658512. doi: 10.3389/fvets.2021.658512 (PMC8263912; doi:10.3389/fvets.2021.658512)
Supplement: Supplementary file 1 [file Data_Sheet_1.pdf]

## *Supplementary Material*

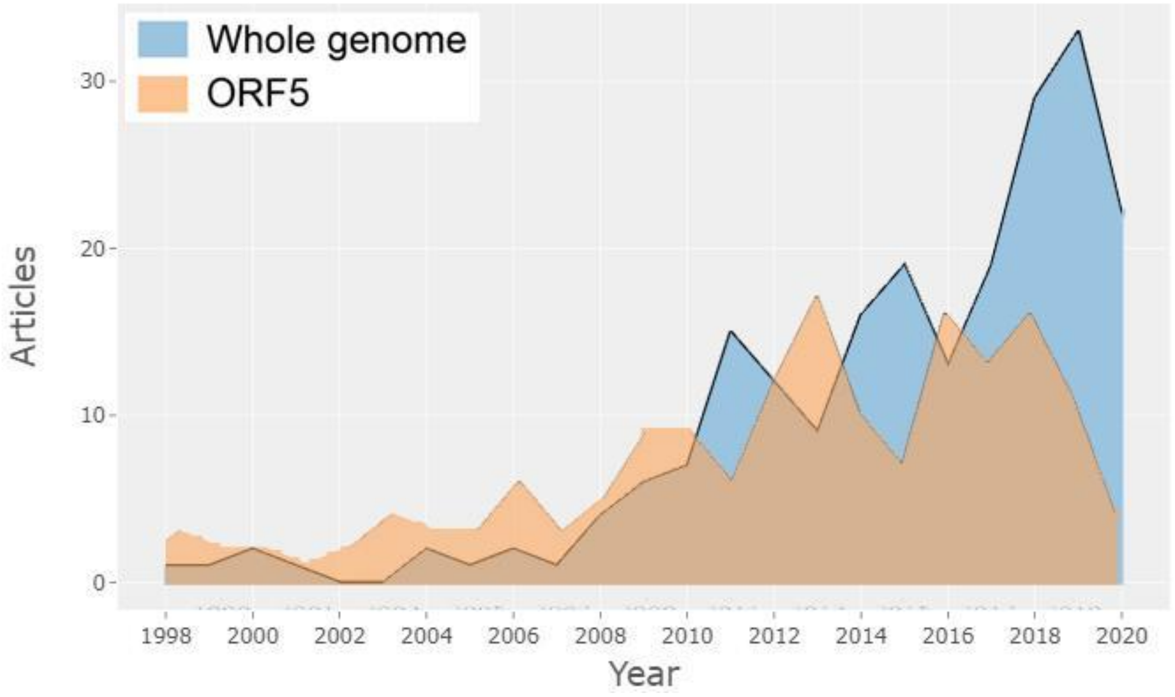


**Supplementary Figure 1.** Comparison of the number of articles per year published for whole genome (blue) and ORF5 (orange) up to the day of our bibliometric search (October 2020).

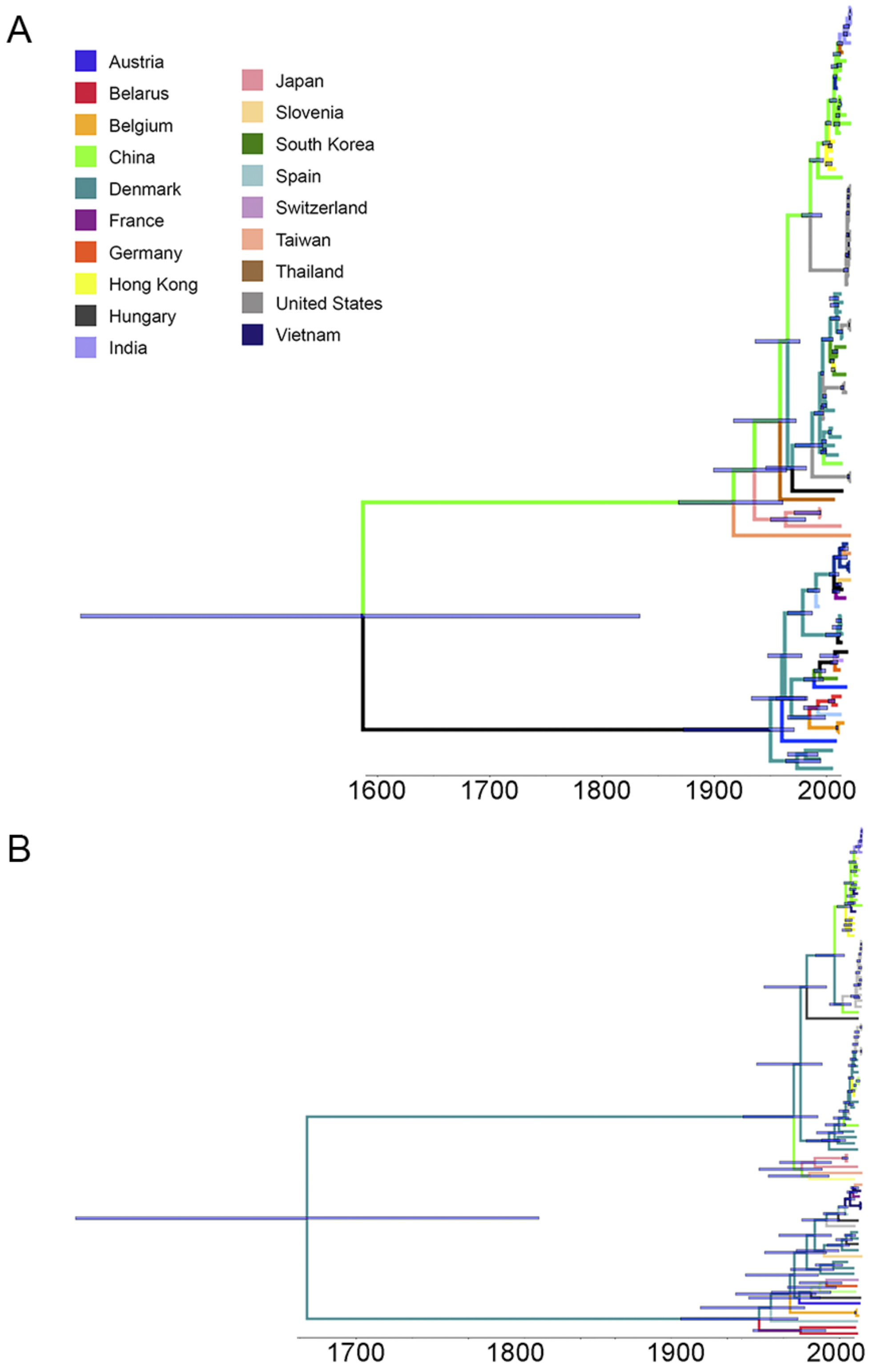


**Supplementary Figure 2.** Comparison of maximum clade credibility phylogenetic trees obtained for A) whole genome and B) ORF-5 databases. Blue bars indicate the 95% highest probability density (HPD) values. Tree branches are colored according to country of origin.

**Supplementary Table 1.** Raw results of the gbmunge R package with the extracted metadata for each genbank ID retrieved for this study**.**

| GenbankID | Length | Submission Date | Country | Collection |
|---|---|---|---|---|
| KT326148 | 15095 | 1/25/2016 | Austria | Nov-13 |
| KT334375 | 15022 | 12/19/2015 | Austria | Mar-14 |
| MT000052 | 15103 | 8/24/2020 | Austria | May-15 |
| JF802085 | 15001 | 2/15/2012 | Belarus | Jan-07 |
| KP889243 | 14953 | 5/28/2015 | Belarus | 2010 |
| KT159249 | 15021 | 11/22/2015 | Belgium | 2013 |
| GU737264 | 15014 | 7/25/2016 | Belgium | 2007 |
| KT159248 | 15033 | 11/22/2015 | Belgium | 2013 |
| MK876228 | 15092 | 11/24/2019 | Belgium | 1996 |
| NC_026439 | 14953 | 8/23/2018 | Cameroon | 26-Apr-10 |
| KP026921 | 14953 | 3/3/2017 | Cameroon | 26-Apr-10 |
| JF748718 | 15337 | 9/25/2012 | China | Nov-10 |
| JF748717 | 15266 | 9/25/2012 | China | 2009 |
| MH370474 | 15108 | 11/5/2018 | China | 12-Mar-18 |
| GQ359108 | 15358 | 7/24/2016 | China | 6-Sep-08 |

| | | | | |
|---|---|---|---|---|
| KC527830 | 15339 | 12/9/2015 | China | Jun-11 |
| KF611905 | 15020 | 7/14/2015 | China | 1-Jan-13 |
| KJ819936 | 15195 | 8/17/2014 | China | 13-Apr-14 |
| KJ819935 | 15339 | 8/17/2014 | China | 13-Apr-14 |
| KJ819934 | 15339 | 8/17/2014 | China | 13-Apr-14 |
| KJ609517 | 15338 | 6/16/2014 | China | 23-Jul-13 |
| KJ609516 | 15338 | 6/16/2014 | China | 23-Jul-13 |
| KJ546412 | 15341 | 5/25/2014 | China | 4-Jul-13 |
| KJ534543 | 15338 | 5/20/2014 | China | 10-Jul-13 |
| KJ534542 | 15338 | 5/20/2014 | China | 10-Jul-13 |
| KJ534541 | 15345 | 5/20/2014 | China | 2-Jun-13 |
| KJ534540 | 15360 | 5/20/2014 | China | 2-Jun-13 |
| KJ534539 | 15345 | 5/20/2014 | China | 2-Jun-13 |
| KJ019330 | 15338 | 3/24/2014 | China | 1-Jun-13 |
| KJ002452 | 15338 | 3/17/2014 | China | 20-Sep-13 |
| KJ002451 | 15352 | 3/17/2014 | China | 30-Jun-13 |
| JQ308798 | 15526 | 8/13/2012 | China | 16-Jan-11 |

| | | | | |
|---|---|---|---|---|
| JN662424 | 15559 | 8/13/2012 | China | Jan-11 |
| JQ743666 | 15526 | 6/29/2012 | China | Oct-10 |
| MH663433 | 15338 | 6/16/2019 | China | 2017 |
| KY761966 | 15320 | 3/26/2017 | China | 22-Jan-16 |
| GU461292 | 15348 | 7/25/2016 | China | 10-Jul-07 |
| GU047345 | 15068 | 7/24/2016 | China | 2009 |
| GU047344 | 15059 | 7/24/2016 | China | 2006 |
| GQ351601 | 15444 | 7/24/2016 | China | Jun-07 |
| KT945018 | 15049 | 5/10/2016 | China | 2015 |
| KT945017 | 15049 | 5/3/2016 | China | 2015 |
| KT358728 | 15271 | 3/1/2016 | China | 2015 |
| KM196101 | 15083 | 10/16/2014 | China | 28-Nov-12 |
| KC445138 | 15260 | 8/8/2013 | China | 17-Sep-12 |
| JX187609 | 15081 | 5/17/2013 | China | 2011 |
| JX878380 | 15350 | 12/19/2012 | China | Aug-11 |
| JX878379 | 15349 | 12/19/2012 | China | Aug-11 |
| JX087437 | 15320 | 8/6/2012 | China | Apr-12 |

| | | | | |
|---|---|---|---|---|
| JQ715698 | 15295 | 5/20/2012 | China | 2011 |
| JQ715697 | 15315 | 5/20/2012 | China | 2011 |
| JQ804986 | 15319 | 5/16/2012 | China | 2009 |
| JQ326271 | 15339 | 4/4/2012 | China | Sep-11 |
| MH651748 | 15013 | 11/4/2018 | China | Jul-16 |
| MH651747 | 15022 | 11/4/2018 | China | Sep-16 |
| MH651746 | 15008 | 11/4/2018 | China | Sep-16 |
| MH651745 | 15015 | 11/4/2018 | China | Jun-16 |
| MH651744 | 15019 | 11/4/2018 | China | Mar-16 |
| MH651743 | 15021 | 11/4/2018 | China | Feb-16 |
| MH651742 | 15021 | 11/4/2018 | China | Apr-16 |
| MH651741 | 15019 | 11/4/2018 | China | Apr-16 |
| MH651740 | 15019 | 11/4/2018 | China | Jun-16 |
| MH651739 | 15014 | 11/4/2018 | China | Apr-16 |
| MH651738 | 15021 | 11/4/2018 | China | Jun-16 |
| MH651737 | 15015 | 11/4/2018 | China | Apr-16 |
| MH651736 | 15021 | 11/4/2018 | China | Apr-16 |

| | | | | |
|---|---|---|---|---|
| MH121061 | 15013 | 8/31/2018 | China | 30-Mar-17 |
| MH068878 | 15019 | 7/25/2018 | China | Jul-17 |
| MH324400 | 15315 | 7/25/2018 | China | 2017 |
| KY373218 | 15344 | 11/13/2017 | China | 16-Oct-14 |
| KX650082 | 15341 | 12/6/2016 | China | 2008 |
| KX766379 | 15040 | 10/23/2016 | China | 2016 |
| KX766378 | 15345 | 10/23/2016 | China | 2007 |
| JN387274 | 15235 | 7/25/2016 | China | 10-Feb-11 |
| JN387273 | 15235 | 7/25/2016 | China | 10-Nov-10 |
| JN387272 | 15339 | 7/25/2016 | China | 22-Jul-10 |
| JN387271 | 15357 | 7/25/2016 | China | 20-Mar-07 |
| GU143913 | 15338 | 7/24/2016 | China | Feb-09 |
| KU978619 | 15304 | 7/16/2016 | China | 12-Oct-15 |
| KP330232 | 15342 | 6/2/2016 | China | 20-Feb-14 |
| KT445876 | 15352 | 2/3/2016 | China | 10-Jul-14 |
| KT351740 | 15439 | 12/21/2015 | China | 12-Jan-13 |
| KT351739 | 15208 | 12/21/2015 | China | 12-Nov-13 |

| | | | | |
|---|---|---|---|---|
| KP861625 | 15020 | 12/17/2015 | China | 30-Aug-14 |
| KC492506 | 15058 | 12/3/2015 | China | 2011 |
| KC492505 | 15058 | 12/3/2015 | China | 2011 |
| KC492504 | 15061 | 12/3/2015 | China | 2011 |
| KJ855518 | 15335 | 10/6/2014 | China | 23-Jan-10 |
| KF771273 | 15441 | 5/25/2014 | China | 1-Oct-11 |
| KJ541663 | 15429 | 5/21/2014 | China | 6-Jun-05 |
| KJ143621 | 15020 | 4/2/2014 | China | 1-Dec-12 |
| GQ499196 | 15373 | 3/8/2013 | China | 2008 |
| GQ499195 | 15373 | 3/8/2013 | China | 2008 |
| GQ499194 | 15373 | 3/8/2013 | China | 2008 |
| GQ499193 | 15373 | 3/8/2013 | China | 2008 |
| JN836553 | 15329 | 11/20/2012 | China | May-09 |
| JX317649 | 15320 | 9/24/2012 | China | Aug-10 |
| MN660070 | 15019 | 5/6/2020 | China | 2018 |
| MN648450 | 15098 | 2/25/2020 | China | Dec-18 |
| MN648449 | 15112 | 2/25/2020 | China | Jan-19 |

| | | | | |
|---|---|---|---|---|
| MN648055 | 15112 | 2/25/2020 | China | Feb-19 |
| MN648054 | 15112 | 2/25/2020 | China | Jun-18 |
| MK396384 | 15019 | 12/31/2019 | China | Sep-18 |
| MK396383 | 15019 | 12/31/2019 | China | Aug-18 |
| MK396382 | 15026 | 12/31/2019 | China | Jul-18 |
| MK396381 | 15010 | 12/31/2019 | China | Jun-18 |
| MK396380 | 15016 | 12/31/2019 | China | Apr-18 |
| MK396379 | 15022 | 12/31/2019 | China | Feb-18 |
| MK396378 | 15031 | 12/31/2019 | China | Nov-17 |
| MK396377 | 15022 | 12/31/2019 | China | Oct-17 |
| MK396376 | 15022 | 12/31/2019 | China | Jul-17 |
| MN046243 | 15004 | 1/3/2020 | China | 2018 |
| MN046242 | 15339 | 1/3/2020 | China | 2018 |
| MN046241 | 15005 | 1/3/2020 | China | 2016 |
| MN046240 | 15005 | 1/3/2020 | China | 2016 |
| MN046239 | 15326 | 1/3/2020 | China | 2018 |
| MN046238 | 15325 | 1/3/2020 | China | 2016 |

| | | | | |
|---|---|---|---|---|
| MN046237 | 15322 | 1/3/2020 | China | 2015 |
| MN046236 | 15321 | 1/3/2020 | China | 2017 |
| MN046235 | 15320 | 1/3/2020 | China | 2017 |
| MN046234 | 15326 | 1/3/2020 | China | 2016 |
| MN046233 | 15320 | 1/3/2020 | China | 2017 |
| MN046232 | 15323 | 1/3/2020 | China | 2018 |
| MN046231 | 15319 | 1/3/2020 | China | 2017 |
| MN046230 | 15019 | 1/3/2020 | China | 2018 |
| MN046229 | 15018 | 1/3/2020 | China | 2018 |
| MN046228 | 15017 | 1/3/2020 | China | 2014 |
| MN046227 | 15014 | 1/3/2020 | China | 2014 |
| MN046226 | 15018 | 1/3/2020 | China | 2014 |
| MN046225 | 15014 | 1/3/2020 | China | 2014 |
| MN046224 | 15021 | 1/3/2020 | China | 2017 |
| MN046223 | 15023 | 1/3/2020 | China | 2018 |
| MN046222 | 15013 | 1/3/2020 | China | 2016 |
| MN046221 | 15013 | 1/3/2020 | China | 2014 |

| | | | | |
|---|---|---|---|---|
| MK906026 | 14960 | 10/27/2019 | China | Dec-17 |
| MK759853 | 14960 | 10/27/2019 | China | Apr-17 |
| MK429987 | 15314 | 7/29/2019 | China | 10-Oct-18 |
| MH500776 | 15013 | 3/2/2019 | China | Oct-17 |
| MH236426 | 15307 | 2/11/2019 | China | 16-Aug-16 |
| MH167388 | 14998 | 10/27/2018 | China | 16-Oct-17 |
| MG687491 | 15027 | 10/20/2018 | China | 12-Sep-17 |
| MG844181 | 15011 | 9/4/2018 | China | 8-Jun-17 |
| MF770574 | 15325 | 7/31/2018 | China | Jan-17 |
| MF689000 | 15320 | 7/24/2018 | China | May-12 |
| MG913987 | 15112 | 7/2/2018 | China | 2017 |
| MG860516 | 15109 | 7/1/2018 | China | 2017 |
| MF375261 | 15012 | 5/20/2018 | China | 30-Oct-15 |
| MF375260 | 15013 | 5/20/2018 | China | 20-Oct-15 |
| MG914067 | 15015 | 5/19/2018 | China | 2017 |
| KY053458 | 15073 | 5/11/2018 | China | 2-Jun-16 |
| MF196906 | 15321 | 4/3/2018 | China | 2016 |

| | | | | |
|---|---|---|---|---|
| MF196905 | 15321 | 4/3/2018 | China | 2016 |
| KX689233 | 15391 | 3/3/2018 | China | 23-Feb-15 |
| MF124329 | 15321 | 7/16/2017 | China | Apr-14 |
| KY412888 | 15016 | 6/10/2017 | China | 2014 |
| KY412887 | 15016 | 6/10/2017 | China | 2014 |
| KY041782 | 15020 | 5/6/2017 | China | Apr-16 |
| KX900392 | 15020 | 5/6/2017 | China | Dec-15 |
| KX815434 | 15327 | 4/17/2017 | China | 2015 |
| KX621003 | 15413 | 4/16/2017 | China | 30-Dec-15 |
| KX767091 | 15357 | 3/8/2017 | China | 2015 |
| KX357708 | 15321 | 2/12/2017 | China | 2016 |
| KM189443 | 15313 | 7/31/2016 | China | 8-Jan-12 |
| JF268684 | 15347 | 7/25/2016 | China | Nov-09 |
| HQ233605 | 15439 | 7/25/2016 | China | 2002 |
| HQ233604 | 15349 | 7/25/2016 | China | 2006 |
| HM011104 | 15352 | 7/25/2016 | China | Jan-07 |
| KU950375 | 15320 | 5/25/2016 | China | Nov-15 |

| | | | | |
|---|---|---|---|---|
| KU950374 | 15017 | 5/25/2016 | China | Oct-15 |
| KU950373 | 15319 | 5/25/2016 | China | Mar-14 |
| KU950372 | 15020 | 5/25/2016 | China | Dec-14 |
| KU950371 | 15023 | 5/25/2016 | China | Apr-15 |
| KU950370 | 15320 | 5/25/2016 | China | Apr-15 |
| KT224385 | 15113 | 12/12/2015 | China | Aug-14 |
| KP890342 | 15340 | 9/8/2015 | China | 19-Feb-13 |
| KJ747052 | 15260 | 8/18/2015 | China | Feb-11 |
| KM453698 | 15326 | 6/10/2015 | China | Feb-14 |
| KM261784 | 15320 | 11/3/2014 | China | Mar-14 |
| KM000066 | 15319 | 10/12/2014 | China | 2-Apr-14 |
| KJ523897 | 15428 | 10/2/2014 | China | 15-Dec-11 |
| KJ523896 | 15430 | 10/2/2014 | China | 9-Feb-10 |
| KJ523895 | 15431 | 10/2/2014 | China | 7-Jun-09 |
| KJ523894 | 15435 | 10/2/2014 | China | 3-Aug-08 |
| KJ591659 | 15336 | 6/21/2014 | China | Sep-13 |
| KF751238 | 15369 | 4/16/2014 | China | 25-Oct-11 |

| | | | | |
|---|---|---|---|---|
| KF751237 | 15344 | 4/16/2014 | China | 13-Oct-11 |
| KF001144 | 15113 | 8/25/2013 | China | 30-Apr-11 |
| JX235370 | 15328 | 7/20/2013 | China | 27-Aug-11 |
| JX215554 | 15327 | 7/20/2013 | China | 9-May-11 |
| JX215553 | 15333 | 7/20/2013 | China | 14-Dec-10 |
| JX215552 | 15333 | 7/20/2013 | China | 8-Feb-10 |
| JX215551 | 15334 | 7/20/2013 | China | 17-Jan-11 |
| JX192639 | 15327 | 7/20/2013 | China | 11-Jan-10 |
| JX192638 | 15329 | 7/20/2013 | China | 6-Sep-10 |
| JX192637 | 15332 | 7/20/2013 | China | 17-Aug-10 |
| JX192636 | 15329 | 7/20/2013 | China | 20-Oct-10 |
| JX192635 | 15333 | 7/20/2013 | China | 10-Oct-10 |
| JX192634 | 15330 | 7/20/2013 | China | 13-Mar-10 |
| JX192633 | 15326 | 7/20/2013 | China | 14-Apr-10 |
| JX192632 | 15327 | 7/20/2013 | China | 7-Jul-10 |
| JX235367 | 15327 | 7/20/2013 | China | 2-Sep-11 |
| JX235366 | 15328 | 7/20/2013 | China | 12-Jul-11 |

| | | | | |
|---|---|---|---|---|
| JX235365 | 15330 | 7/20/2013 | China | 16-May-11 |
| JX217036 | 15327 | 7/20/2013 | China | 20-Mar-11 |
| KC422731 | 15319 | 2/25/2013 | China | 2009 |
| JX177644 | 15324 | 9/1/2012 | China | Apr-12 |
| MN862433 | 15046 | 7/30/2020 | China | 2018 |
| MN550991 | 15026 | 6/27/2020 | China | Jun-18 |
| MN606305 | 15409 | 5/27/2020 | China | 29-Jun-07 |
| MN606304 | 15014 | 5/27/2020 | China | 10-Dec-18 |
| MT379661 | 15320 | 5/13/2020 | China | 2018 |
| MN660069 | 15018 | 5/6/2020 | China | 2014 |
| MN660068 | 15265 | 5/6/2020 | China | 6-Sep-13 |
| MN660067 | 15265 | 5/6/2020 | China | 6-Sep-13 |
| MK780825 | 15431 | 3/4/2020 | China | Aug-16 |
| MK780824 | 15445 | 3/4/2020 | China | Oct-17 |
| MK450333 | 15020 | 8/19/2019 | China | 2018-10 |
| MK303390 | 14932 | 7/6/2019 | China | 1-Sep-18 |
| MK214314 | 14975 | 6/18/2019 | China | 1-May-15 |

| | | | | |
|---|---|---|---|---|
| MK202794 | 15112 | 6/17/2019 | China | 2018 |
| MK450365 | 15347 | 6/12/2019 | China | Aug-18 |
| MK144543 | 15014 | 6/12/2019 | China | 2018 |
| MK144542 | 15263 | 6/12/2019 | China | 2017 |
| MH588710 | 15019 | 3/24/2019 | China | 2016 |
| KX967492 | 15092 | 10/31/2018 | China | 2015 |
| MF669722 | 15346 | 10/8/2018 | China | 23-Dec-14 |
| MF669721 | 15320 | 10/8/2018 | China | 17-Jan-15 |
| MF669720 | 14988 | 10/8/2018 | China | 2-Apr-14 |
| MF766474 | 15005 | 7/25/2018 | China | 20-May-16 |
| MF766473 | 15320 | 7/25/2018 | China | 25-May-15 |
| MF766472 | 15320 | 7/25/2018 | China | 25-May-15 |
| MF766471 | 15003 | 7/25/2018 | China | 4-Jan-14 |
| MF766470 | 15317 | 7/25/2018 | China | 28-Jul-13 |
| MH078490 | 15010 | 7/25/2018 | China | 2017 |
| MF818049 | 15321 | 7/23/2018 | China | 2016 |
| KX980392 | 15367 | 5/11/2018 | China | Dec-15 |

| | | | | |
|---|---|---|---|---|
| MG011719 | 15024 | 4/14/2018 | China | 2017 |
| MG011718 | 15017 | 4/14/2018 | China | 2017 |
| KY745901 | 15304 | 2/14/2018 | China | 2016 |
| MF370557 | 15319 | 1/9/2018 | China | Sep-06 |
| KY290748 | 15018 | 6/19/2017 | China | Mar-16 |
| KR706343 | 15019 | 8/23/2015 | China | 2013 |
| KX169191 | 15017 | 11/23/2016 | China | Apr-14 |
| JF800911 | 15248 | 7/25/2016 | China | 25-Dec-09 |
| KU523367 | 15019 | 7/23/2016 | China | 21-Sep-11 |
| HM189676 | 15326 | 7/25/2016 | China | May-09 |
| GU168569 | 15345 | 7/24/2016 | China | 26-Aug-08 |
| GU168568 | 15353 | 7/24/2016 | China | 10-Mar-09 |
| GQ374442 | 15338 | 7/24/2016 | China | 2008 |
| GQ374441 | 15339 | 7/24/2016 | China | 2007 |
| GU232738 | 15311 | 7/24/2016 | China | 14-Nov-08 |
| GU232735 | 15301 | 7/24/2016 | China | 8-Dec-08 |
| KU201579 | 15353 | 5/9/2016 | China | 2008 |

| | | | | |
|---|---|---|---|---|
| KT819203 | 15239 | 3/2/2016 | China | 14-Dec-14 |
| KT022072 | 15348 | 1/31/2016 | China | 2013 |
| KT022071 | 15348 | 1/31/2016 | China | 2014 |
| KP742987 | 14989 | 1/31/2016 | China | 2014 |
| KP742986 | 14988 | 1/31/2016 | China | 2014 |
| KT180169 | 15220 | 11/24/2015 | China | 29-Nov-13 |
| KT033733 | 15349 | 11/18/2015 | China | 2008 |
| KP771784 | 15307 | 9/15/2015 | China | 2014 |
| KP771783 | 14962 | 9/15/2015 | China | 2014 |
| KP771782 | 15323 | 9/15/2015 | China | 2014 |
| KP771781 | 15322 | 9/15/2015 | China | 2014 |
| KP771780 | 15322 | 9/15/2015 | China | 2014 |
| KP771779 | 15281 | 9/15/2015 | China | 2008 |
| KP771778 | 15354 | 9/15/2015 | China | 2011 |
| KP771777 | 15322 | 9/15/2015 | China | 2012 |
| KP771776 | 15322 | 9/15/2015 | China | 2012 |
| KP771775 | 15322 | 9/15/2015 | China | 2012 |

| | | | | |
|---|---|---|---|---|
| KP771774 | 15322 | 9/15/2015 | China | 2011 |
| KP771773 | 15322 | 9/15/2015 | China | 2012 |
| KP771772 | 15322 | 9/15/2015 | China | 2012 |
| KP771771 | 15322 | 9/15/2015 | China | 2012 |
| KP771770 | 15322 | 9/15/2015 | China | 2012 |
| KP771769 | 15322 | 9/15/2015 | China | 2012 |
| KP771768 | 15322 | 9/15/2015 | China | 2012 |
| KP771767 | 15322 | 9/15/2015 | China | 2011 |
| KP771766 | 15322 | 9/15/2015 | China | 2011 |
| KP771765 | 15322 | 9/15/2015 | China | 2011 |
| KP771764 | 15322 | 9/15/2015 | China | 2012 |
| KP771763 | 15322 | 9/15/2015 | China | 2012 |
| KP771762 | 15322 | 9/15/2015 | China | 2012 |
| KP771761 | 15322 | 9/15/2015 | China | 2012 |
| KP771760 | 15322 | 9/15/2015 | China | 2012 |
| KP771759 | 15322 | 9/15/2015 | China | 2012 |
| KP771758 | 15322 | 9/15/2015 | China | 2012 |

| | | | | |
|---|---|---|---|---|
| KP771757 | 15322 | 9/15/2015 | China | 2012 |
| KP771756 | 15322 | 9/15/2015 | China | 2012 |
| KP771755 | 15322 | 9/15/2015 | China | 2008 |
| KP771754 | 15322 | 9/15/2015 | China | 2008 |
| KP771753 | 15322 | 9/15/2015 | China | 2013 |
| KP771752 | 15322 | 9/15/2015 | China | 2013 |
| KP771751 | 15322 | 9/15/2015 | China | 2013 |
| KP771750 | 15322 | 9/15/2015 | China | 2013 |
| KP771749 | 15322 | 9/15/2015 | China | 2011 |
| KP771748 | 15322 | 9/15/2015 | China | 2011 |
| KP771747 | 15322 | 9/15/2015 | China | 2012 |
| KP771746 | 15322 | 9/15/2015 | China | 2011 |
| KP771745 | 15178 | 9/15/2015 | China | 2013 |
| KP771744 | 15178 | 9/15/2015 | China | 2013 |
| KP771743 | 15322 | 9/15/2015 | China | 2013 |
| KP771742 | 15322 | 9/15/2015 | China | 2013 |
| KP771741 | 15322 | 9/15/2015 | China | 2013 |

| | | | | |
|---|---|---|---|---|
| KP771740 | 15322 | 9/15/2015 | China | 2013 |
| KP771739 | 15322 | 9/15/2015 | China | 2014 |
| KP771738 | 15322 | 9/15/2015 | China | 2014 |
| KP771737 | 15322 | 9/15/2015 | China | 2014 |
| KP771736 | 15322 | 9/15/2015 | China | 2014 |
| KP771735 | 15322 | 9/15/2015 | China | 2014 |
| KP780882 | 15045 | 8/12/2015 | China | 12-Nov-14 |
| KP780881 | 15045 | 8/12/2015 | China | 15-Oct-14 |
| KP793736 | 15332 | 8/10/2015 | China | 9-Jun-11 |
| KM453699 | 15326 | 12/13/2014 | China | Feb-14 |
| JX679179 | 15429 | 11/18/2012 | China | 2011 |
| JQ663568 | 15347 | 6/20/2012 | China | 2010 |
| JQ663567 | 15347 | 6/20/2012 | China | 2010 |
| JQ663566 | 15347 | 6/20/2012 | China | 2010 |
| JQ663565 | 15347 | 6/20/2012 | China | 2010 |
| JQ663564 | 15347 | 6/20/2012 | China | 2010 |
| JQ663563 | 15347 | 6/20/2012 | China | 2010 |

| | | | | |
|---|---|---|---|---|
| JQ663562 | 15347 | 6/20/2012 | China | 2010 |
| JQ663561 | 15347 | 6/20/2012 | China | 2010 |
| JQ663560 | 15347 | 6/20/2012 | China | 2010 |
| JQ663559 | 15347 | 6/20/2012 | China | 2010 |
| JQ663558 | 15347 | 6/20/2012 | China | 2010 |
| JQ663557 | 15347 | 6/20/2012 | China | 2010 |
| JQ663556 | 15347 | 6/20/2012 | China | 2010 |
| JQ663555 | 15347 | 6/20/2012 | China | 2010 |
| JQ663554 | 15352 | 6/20/2012 | China | 2012 |
| JQ663553 | 15352 | 6/20/2012 | China | 2010 |
| JQ663552 | 15344 | 6/20/2012 | China | 2010 |
| JQ663551 | 15348 | 6/20/2012 | China | 2010 |
| JQ663550 | 15350 | 6/20/2012 | China | 2010 |
| JQ663549 | 15347 | 6/20/2012 | China | 2010 |
| JQ663548 | 15347 | 6/20/2012 | China | 2010 |
| JQ663547 | 15347 | 6/20/2012 | China | 2010 |
| JQ663546 | 15347 | 6/20/2012 | China | 2010 |

| | | | | |
|---|---|---|---|---|
| JQ663545 | 15347 | 6/20/2012 | China | 2010 |
| JQ663544 | 15347 | 6/20/2012 | China | 2010 |
| JQ663543 | 15352 | 6/20/2012 | China | 2010 |
| JQ663542 | 15347 | 6/20/2012 | China | 2010 |
| JQ663541 | 15347 | 6/20/2012 | China | 2010 |
| JQ663540 | 15347 | 6/20/2012 | China | 2010 |
| GU168567 | 15332 | 12/6/2009 | China | 10-Mar-09 |
| JN864948 | 15442 | 2/29/2012 | China | 2007 |
| EU860249 | 15356 | 11/21/2012 | China | Mar-07 |
| EU860248 | 15324 | 4/25/2012 | China | Oct-06 |
| FJ536165 | 15434 | 7/24/2016 | China | 2004 |
| FJ899592 | 15445 | 7/24/2016 | China | 2003 |
| EU708726 | 15373 | 7/26/2016 | China | 9-Dec-06 |
| FJ889130 | 15344 | 7/24/2016 | China | Aug-08 |
| FJ889129 | 15353 | 7/24/2016 | China | Oct-08 |
| EF488048 | 15373 | 7/22/2016 | China | 4-Nov-07 |
| HM853673 | 15347 | 3/12/2013 | China | Sep-08 |

| | | | | |
|---|---|---|---|---|
| GU169411 | 15320 | 12/2/2009 | China | 16-Jan-08 |
| FJ950747 | 15345 | 5/16/2009 | China | 17-Feb-07 |
| FJ393459 | 15340 | 7/26/2016 | China | Aug-07 |
| FJ393458 | 15347 | 7/26/2016 | China | Jul-07 |
| FJ393457 | 15347 | 7/26/2016 | China | May-07 |
| FJ393456 | 15347 | 7/26/2016 | China | Jan-07 |
| EU939312 | 15351 | 7/26/2016 | China | 12-Jul-06 |
| EU864233 | 15346 | 7/26/2016 | China | 11-Nov-06 |
| EU864232 | 15442 | 7/26/2016 | China | 20-Jan-05 |
| EU864231 | 15234 | 7/26/2016 | China | 6-Sep-07 |
| MK639926 | 15064 | 6/30/2020 | China | May-18 |
| MN642105 | 15315 | 6/21/2020 | China | Nov-14 |
| MN642104 | 15412 | 6/21/2020 | China | Feb-17 |
| MN642103 | 14960 | 6/21/2020 | China | Dec-16 |
| MN642102 | 15315 | 6/21/2020 | China | Dec-16 |
| MN642101 | 15013 | 6/21/2020 | China | Dec-14 |
| MN642100 | 15318 | 6/21/2020 | China | Nov-14 |

| | | | | |
|---|---|---|---|---|
| MN642099 | 15429 | 6/21/2020 | China | Mar-15 |
| MK453050 | 15110 | 12/11/2019 | China | 2018 |
| MK453049 | 15093 | 12/11/2019 | China | 2018 |
| NC_040535 | 15224 | 9/12/2019 | China | Dec-14 |
| KY369969 | 14943 | 11/20/2018 | China | Dec-15 |
| KY369968 | 15275 | 11/20/2018 | China | Dec-14 |
| KY369967 | 15224 | 11/20/2018 | China | Dec-14 |
| MH191378 | 1767 | 8/31/2018 | China | 30-Mar-17 |
| MH121060 | 2000 | 8/31/2018 | China | 30-Mar-17 |
| EU825723 | 15340 | 7/26/2016 | China: Beijing | Mar-07 |
| JF796180 | 15357 | 7/25/2016 | China: Foshan | 11-Nov-10 |
| GU269541 | 15421 | 7/24/2016 | China: GuangDong | Dec-05 |
| EU825724 | 15336 | 7/26/2016 | China: Guangdong | Apr-07 |
| EU624117 | 15326 | 3/28/2012 | China: Guangdong, Jiangmen, Xinhui | May-07 |
| MF526896 | 15309 | 6/18/2018 | China: Guangdong, Qingyuan | 26-Sep-16 |
| HQ315835 | 15361 | 5/3/2013 | China: Guangxi | Jul-09 |

| | | | | |
|---|---|---|---|---|
| JX912249 | 15332 | 1/12/2013 | China: Guangxi | 2010 |
| HQ843179 | 15340 | 7/25/2016 | China: Henan | Apr-09 |
| HQ843178 | 15338 | 7/25/2016 | China: Henan | Apr-09 |
| FJ797690 | 15334 | 7/24/2016 | China: HuNan Province | 14-Sep-06 |
| MN547967 | 14930 | 6/29/2020 | China: Jiangsu | 7-Jul-19 |
| NC_028963 | 15728 | 12/24/2018 | China: Jilin | Aug-14 |
| KP280006 | 15728 | 11/20/2018 | China: Jilin | Aug-14 |
| JX880029 | 14915 | 12/4/2012 | China: Nanjing | 12-Jun-11 |
| HQ315836 | 15439 | 10/2/2014 | China: Nantong | Jan-08 |
| GU454850 | 15274 | 7/25/2016 | China: Qingyuan, Guangdong | 2007 |
| HM016158 | 15320 | 7/25/2016 | China: Shandong, Binzhou | 12-Apr-08 |
| HM016159 | 15320 | 7/25/2016 | China: Shandong, Jinan | 24-Nov-09 |
| HQ843180 | 15345 | 7/25/2016 | China: Shangdong | Apr-09 |
| MF187956 | 15350 | 11/13/2017 | China: Shanghai | 2006 |
| KF678434 | 15313 | 4/9/2014 | China: Shanghai | Dec-12 |

| | | | | |
|---|---|---|---|---|
| HQ843181 | 15337 | 7/25/2016 | China: Shanxi | Jan-09 |
| FJ895329 | 15351 | 7/24/2016 | China: Shanxi province | 11-Feb-09 |
| HQ315837 | 15369 | 7/25/2016 | China: Siyang | Sep-09 |
| MN119309 | 15012 | 2/26/2020 | China: Xinjiang | 1-Apr-19 |
| KF815525 | 14987 | 8/20/2015 | China: Xinjiang, Urumqi | 23-Aug-12 |
| MH046842 | 15323 | 10/16/2018 | China:Fujian | 2017 |
| MH046843 | 15321 | 10/16/2018 | China:Guangdong | 2016 |
| HQ401282 | 15320 | 7/25/2016 | China:Shaanxi | 27-Aug-07 |
| JX857698 | 15432 | 3/13/2013 | China:Yunnan province | 2011 |
| KF183947 | 15402 | 5/14/2014 | Denmark | 2011 |
| KF183946 | 15402 | 5/14/2014 | Denmark | 2010 |
| KC862585 | 15411 | 12/13/2013 | Denmark | 2004 |
| KC862584 | 15411 | 12/13/2013 | Denmark | 2003 |
| KC862583 | 15402 | 12/13/2013 | Denmark | 2010 |
| KC862582 | 15411 | 12/13/2013 | Denmark | 2008 |
| KC862581 | 15399 | 12/13/2013 | Denmark | 2010 |

| | | | | |
|---|---|---|---|---|
| KC862580 | 15402 | 12/13/2013 | Denmark | 2010 |
| KC862579 | 15345 | 12/13/2013 | Denmark | 2010 |
| KC862578 | 15411 | 12/13/2013 | Denmark | 2004 |
| KC862577 | 15411 | 12/13/2013 | Denmark | 2011 |
| KC862576 | 15399 | 12/13/2013 | Denmark | 1997 |
| KC862575 | 15402 | 12/13/2013 | Denmark | 2012 |
| KC862574 | 14889 | 12/13/2013 | Denmark | 2012 |
| KC862573 | 14889 | 12/13/2013 | Denmark | 2008 |
| KC862572 | 15087 | 12/13/2013 | Denmark | 2003 |
| KC862571 | 15102 | 12/13/2013 | Denmark | 2003 |
| KC862569 | 15111 | 12/13/2013 | Denmark | 2011 |
| KC862568 | 15099 | 12/13/2013 | Denmark | 2010 |
| KC862567 | 15109 | 12/13/2013 | Denmark | 2011 |
| KC862566 | 15098 | 12/13/2013 | Denmark | 1992 |
| MN603982 | 15098 | 3/25/2020 | Denmark | 7/31/2019 |
| KY767026 | 15093 | 4/18/2017 | France | Dec-14 |
| KY366411 | 15160 | 3/21/2017 | France | Nov-05 |

| | | | | |
|---|---|---|---|---|
| MN604234 | 15098 | 4/20/2020 | France | Mar-14 |
| MH018883 | 15111 | 11/26/2018 | France | Dec-16 |
| KT344816 | 15095 | 12/19/2015 | Germany | 2009 |
| KF287142 | 15033 | 4/10/2014 | Hong Kong | 1-Jan-04 |
| KF287137 | 15477 | 4/10/2014 | Hong Kong | 1-Jan-04 |
| KF287135 | 15477 | 4/10/2014 | Hong Kong | 1-Jan-04 |
| KF287133 | 15477 | 4/10/2014 | Hong Kong | 1-Jan-03 |
| KF287143 | 15418 | 4/10/2014 | Hong Kong | 1-Jan-04 |
| KF287141 | 15369 | 4/10/2014 | Hong Kong | 1-Jan-04 |
| KF287138 | 15033 | 4/10/2014 | Hong Kong | 1-Jan-04 |
| KF287131 | 14926 | 4/10/2014 | Hong Kong | 1-Jan-04 |
| KF287130 | 14926 | 4/10/2014 | Hong Kong | 1-Jan-04 |
| KF287129 | 14926 | 4/10/2014 | Hong Kong | 1-Jan-03 |
| KF287128 | 14842 | 4/10/2014 | Hong Kong | 1-Jan-04 |
| KF287140 | 15368 | 9/16/2013 | Hong Kong | 1-Jan-05 |
| KF287139 | 15369 | 9/16/2013 | Hong Kong | 1-Jan-04 |
| KF287136 | 15217 | 9/16/2013 | Hong Kong | 1-Jan-04 |

| | | | | |
|---|---|---|---|---|
| KF287134 | 15283 | 9/16/2013 | Hong Kong | 1-Jan-03 |
| KF287132 | 15365 | 9/16/2013 | Hong Kong | 1-Jan-03 |
| KM514315 | 15383 | 11/12/2014 | Hungary | 2012 |
| KJ415276 | 15098 | 11/12/2014 | Hungary | 2012 |
| MK167464 | 15081 | 8/12/2019 | Hungary | 2016 |
| KR296711 | 15099 | 10/11/2015 | Hungary | 2011 |
| MH463458 | 15103 | 2/20/2019 | Hungary: Gyor-Moson-Sopron country | 11-Mar-16 |
| MH463457 | 15111 | 2/20/2019 | Hungary: Gyor-Moson-Sopron country | 11-Mar-16 |
| MH463456 | 15069 | 2/20/2019 | Hungary: Gyor-Moson-Sopron country | 11-Mar-16 |
| MH463455 | 15000 | 2/20/2019 | Hungary: Gyor-Moson-Sopron country | 11-Mar-16 |
| MH463459 | 15113 | 2/20/2019 | Hungary: Komarom-Esztergom country | 2-May-16 |
| MK315210 | 15352 | 7/23/2019 | India | 19-Apr-18 |
| MK315209 | 15352 | 7/23/2019 | India | 10-Apr-18 |
| MK315208 | 15352 | 7/23/2019 | India | 2-Apr-18 |
| MK287895 | 15352 | 7/20/2019 | India | 11-Apr-18 |

| | | | | |
|---|---|---|---|---|
| MK287894 | 15352 | 7/20/2019 | India | 5-Apr-18 |
| AB811789 | 15284 | 5/13/2015 | Japan: Aomori | 2010-08 |
| AB811787 | 15304 | 5/13/2015 | Japan: Aomori | 2000 |
| AB811785 | 15311 | 5/13/2015 | Japan: Chiba | 1992 |
| AB811786 | 15313 | 5/13/2015 | Japan: Nagasaki | 2011-07 |
| AB811788 | 15308 | 5/13/2015 | Japan: Yamagata | 2010-11 |
| AB288356 | 15401 | 6/25/2008 | Japan:Chiba | 1992 |
| JN626287 | 15337 | 7/25/2016 | Laos | 2010 |
| KJ127878 | 14758 | 5/15/2014 | Netherlands | 1-Jul-99 |
| KX668221 | 15049 | 5/8/2019 | Russia | 2013 |
| MT008024 | 14941 | 8/11/2020 | Russia | 2016 |
| MK814111 | 606 | 11/12/2019 | Slovenia | 21-Feb-18 |
| KT033457 | 15065 | 11/5/2015 | South Korea | 4-Mar-07 |
| MT178233 | 14989 | 8/3/2020 | South Korea | 2019 |
| MT176434 | 14989 | 8/3/2020 | South Korea | 2019 |
| KY363992 | 15018 | 12/28/2016 | South Korea | 2016 |
| KY363991 | 15018 | 12/28/2016 | South Korea | 2016 |

| | | | | |
|---|---|---|---|---|
| KU512805 | 15018 | 4/9/2016 | South Korea | 2015 |
| KU512804 | 15018 | 4/9/2016 | South Korea | 2015 |
| KU512803 | 15018 | 4/9/2016 | South Korea | 2015 |
| KU512802 | 15018 | 4/9/2016 | South Korea | 2015 |
| KU512801 | 15018 | 4/9/2016 | South Korea | 2015 |
| KU512800 | 15018 | 4/9/2016 | South Korea | 2015 |
| KU512799 | 15018 | 4/9/2016 | South Korea | 2015 |
| KU512798 | 15018 | 4/9/2016 | South Korea | 2015 |
| KU512797 | 15018 | 4/9/2016 | South Korea | 2015 |
| KU512796 | 15018 | 4/9/2016 | South Korea | 2015 |
| KF555451 | 15019 | 4/30/2014 | South Korea | 2013 |
| KF555450 | 15018 | 4/30/2014 | South Korea | 2013 |
| KP256233 | 15095 | 6/14/2016 | South Korea | 8-Dec-14 |
| KM386622 | 15115 | 6/14/2016 | South Korea | 21-Aug-14 |
| KY434183 | 14978 | 9/2/2017 | South Korea | 2016 |
| FJ349261 | 15038 | 7/26/2016 | South Korea | 2007 |
| JX138236 | 14961 | 12/31/2013 | South Korea | 2010 |

| | | | | |
|---|---|---|---|---|
| JX138235 | 15344 | 12/31/2013 | South Korea | 2007 |
| JX138234 | 14965 | 12/31/2013 | South Korea | 2009 |
| JX138233 | 14959 | 12/31/2013 | South Korea | 2007 |
| MK057532 | 14970 | 3/6/2019 | South Korea | 2017 |
| KF203132 | 15111 | 3/10/2014 | Spain | 1991 |
| KC862570 | 15111 | 12/13/2013 | Spain | 1991 |
| KF666950 | 606 | 3/11/2014 | Spain | 22-Oct-10 |
| KX622783 | 15086 | 12/6/2016 | Switzerland | 2012 |
| KP998431 | 15140 | 7/28/2015 | Taiwan | 1991 |
| MN401750 | 14966 | 4/14/2020 | Taiwan | Jul-18 |
| MN242825 | 15111 | 3/31/2020 | Taiwan: Yunlin | 10/23/2018 |
| KF735060 | 15320 | 5/21/2015 | Thailand | 2010 |
| MK774670 | 3188 | 6/12/2019 | Thailand: Chonburi | 2012 |
| MK774669 | 3188 | 6/12/2019 | Thailand: Ratchaburi | 2004 |
| KU560579 | 14923 | 3/7/2016 | United Kingdom | 2005 |
| KX462792 | 15470 | 7/22/2017 | USA | 23-Apr-12 |
| KU318406 | 14927 | 1/11/2017 | USA | Apr-15 |

| | | | | |
|---|---|---|---|---|
| KT988004 | 14843 | 12/15/2015 | USA | 2006 |
| KT581982 | 15046 | 9/8/2015 | USA | 2-Jun-14 |
| KC469618 | 15432 | 9/16/2013 | USA | 1995 |
| JN660150 | 14968 | 10/12/2012 | USA | 2008 |
| JN654459 | 15020 | 10/12/2012 | USA | 2008 |
| JN654458 | 15412 | 10/12/2012 | USA | 1996 |
| MN073180 | 14919 | 1/3/2020 | USA | 1-Feb-18 |
| MN073178 | 14971 | 1/3/2020 | USA | 18-Aug-17 |
| MN073177 | 14971 | 1/3/2020 | USA | 18-Aug-17 |
| MN073173 | 14908 | 1/3/2020 | USA | 1-Jan-18 |
| MN073172 | 15019 | 1/3/2020 | USA | 15-Jul-18 |
| MN073171 | 15366 | 1/3/2020 | USA | 12-Dec-17 |
| MN073170 | 15395 | 1/3/2020 | USA | 12-Dec-17 |
| MN073169 | 15395 | 1/3/2020 | USA | 18-Sep-17 |
| MN073168 | 15395 | 1/3/2020 | USA | 28-Dec-16 |
| MN073165 | 15395 | 1/3/2020 | USA | 12-Dec-17 |
| MN073164 | 15395 | 1/3/2020 | USA | 12-May-17 |

| | | | | |
|---|---|---|---|---|
| MN073162 | 15395 | 1/3/2020 | USA | 12-Dec-17 |
| MN073161 | 15395 | 1/3/2020 | USA | 12-Dec-17 |
| MN073160 | 15318 | 1/3/2020 | USA | 12-May-17 |
| MN073159 | 15362 | 1/3/2020 | USA | 12-May-17 |
| MN073158 | 15395 | 1/3/2020 | USA | 1-Apr-17 |
| MN073157 | 15379 | 1/3/2020 | USA | 4-Jan-18 |
| MN073155 | 15413 | 1/3/2020 | USA | 4-Nov-16 |
| MN073151 | 14642 | 1/3/2020 | USA | 26-Jan-17 |
| MN073150 | 15071 | 1/3/2020 | USA | 26-Jan-17 |
| MN073149 | 15373 | 1/3/2020 | USA | 26-Jan-17 |
| MN073148 | 15398 | 1/3/2020 | USA | 26-Jan-17 |
| MN073147 | 15411 | 1/3/2020 | USA | 25-May-17 |
| MN073146 | 15356 | 1/3/2020 | USA | 10-May-17 |
| MN073145 | 15386 | 1/3/2020 | USA | 10-May-17 |
| MN073144 | 15327 | 1/3/2020 | USA | 10-May-17 |
| MN073143 | 15389 | 1/3/2020 | USA | 10-May-17 |
| MN073142 | 15353 | 1/3/2020 | USA | 10-May-17 |

| | | | | |
|---|---|---|---|---|
| MN073141 | 15115 | 1/3/2020 | USA | 10-May-17 |
| MN073140 | 15361 | 1/3/2020 | USA | 12-May-17 |
| MN073139 | 15400 | 1/3/2020 | USA | 28-Dec-16 |
| MN073138 | 15106 | 1/3/2020 | USA | 28-Dec-16 |
| MN073137 | 15411 | 1/3/2020 | USA | 12-Aug-17 |
| MN073136 | 15411 | 1/3/2020 | USA | 12-Aug-17 |
| MN073133 | 15387 | 1/3/2020 | USA | 28-Dec-16 |
| MN073132 | 15382 | 1/3/2020 | USA | 6-Feb-18 |
| MN073131 | 15397 | 1/3/2020 | USA | 10-Apr-18 |
| MN073130 | 15402 | 1/3/2020 | USA | 1-Jul-18 |
| MN073129 | 15515 | 1/3/2020 | USA | 4-Jan-18 |
| MN073127 | 15525 | 1/3/2020 | USA | 1-Jul-18 |
| MN073124 | 15001 | 1/3/2020 | USA | 12-May-17 |
| MN073123 | 15001 | 1/3/2020 | USA | 12-May-17 |
| MN073122 | 14980 | 1/3/2020 | USA | 1-Jul-18 |
| MN073121 | 14828 | 1/3/2020 | USA | 11-Oct-17 |
| MN073120 | 15001 | 1/3/2020 | USA | 11-Oct-17 |

| | | | | |
|---|---|---|---|---|
| MN073119 | 15008 | 1/3/2020 | USA | 11-Oct-17 |
| MN073118 | 14993 | 1/3/2020 | USA | 26-Jan-17 |
| MN073117 | 14936 | 1/3/2020 | USA | 26-Jan-17 |
| MN073116 | 14940 | 1/3/2020 | USA | 10-Feb-17 |
| MN073115 | 15015 | 1/3/2020 | USA | 28-Dec-16 |
| MN073114 | 15012 | 1/3/2020 | USA | 28-Dec-16 |
| MN073113 | 15002 | 1/3/2020 | USA | 12-May-17 |
| MN073112 | 15013 | 1/3/2020 | USA | 27-Jan-17 |
| MN073111 | 15013 | 1/3/2020 | USA | 26-Jan-17 |
| MN073102 | 14952 | 1/3/2020 | USA | 1-Feb-18 |
| MN073100 | 14897 | 1/3/2020 | USA | 12-May-17 |
| MN073099 | 14908 | 1/3/2020 | USA | 12-May-17 |
| MN073098 | 14944 | 1/3/2020 | USA | 12-May-17 |
| MN073097 | 14944 | 1/3/2020 | USA | 29-Nov-17 |
| MN073095 | 15105 | 1/3/2020 | USA | 1-Apr-17 |
| MN073092 | 15110 | 1/3/2020 | USA | 12-Dec-17 |
| MN073089 | 15016 | 1/3/2020 | USA | 31-Oct-17 |

| | | | | |
|---|---|---|---|---|
| MN073088 | 15071 | 1/3/2020 | USA | 2-Oct-17 |
| MN073086 | 15071 | 1/3/2020 | USA | 2-Oct-17 |
| MN073084 | 15038 | 1/3/2020 | USA | 23-Mar-18 |
| MN073083 | 14951 | 1/3/2020 | USA | 1-Jul-18 |
| MN073082 | 15027 | 1/3/2020 | USA | 1-Jun-18 |
| MN073081 | 15059 | 1/3/2020 | USA | 7-Jul-18 |
| MK796165 | 14987 | 12/6/2019 | USA | 2018 |
| MK796164 | 14980 | 12/6/2019 | USA | 2018 |
| MK359284 | 15018 | 12/2/2019 | USA | 2018 |
| MK359283 | 15027 | 12/2/2019 | USA | 2018 |
| MK359282 | 14994 | 12/2/2019 | USA | 2018 |
| MK359281 | 14999 | 12/2/2019 | USA | 2018 |
| MK359280 | 15005 | 12/2/2019 | USA | 2018 |
| MK359279 | 14998 | 12/2/2019 | USA | 2017 |
| MK359278 | 14984 | 12/2/2019 | USA | 2017 |
| MK359277 | 14997 | 12/2/2019 | USA | 2017 |
| MK359276 | 15013 | 12/2/2019 | USA | 2017 |

| | | | | |
|---|---|---|---|---|
| MK359275 | 15022 | 12/2/2019 | USA | 2017 |
| MK359274 | 15001 | 12/2/2019 | USA | 2017 |
| MK359273 | 15016 | 12/2/2019 | USA | 2017 |
| MK359272 | 15002 | 12/2/2019 | USA | 2017 |
| MK359271 | 14998 | 12/2/2019 | USA | 2017 |
| MK359270 | 14999 | 12/2/2019 | USA | 2017 |
| MK359269 | 15002 | 12/2/2019 | USA | 2017 |
| MK359268 | 15037 | 12/2/2019 | USA | 2017 |
| MK359267 | 15020 | 12/2/2019 | USA | 2017 |
| MK359266 | 15013 | 12/2/2019 | USA | 2017 |
| MK359265 | 14997 | 12/2/2019 | USA | 2017 |
| MK359264 | 14966 | 12/2/2019 | USA | 2017 |
| MK359263 | 15041 | 12/2/2019 | USA | 2015 |
| MK359262 | 14986 | 12/2/2019 | USA | 2013 |
| MK359261 | 14900 | 12/2/2019 | USA | 2012 |
| MK359260 | 14995 | 12/2/2019 | USA | 2012 |
| MK359259 | 14988 | 12/2/2019 | USA | 2011 |

| | | | | |
|---|---|---|---|---|
| MK359258 | 15033 | 12/2/2019 | USA | 2010 |
| KY348853 | 15436 | 3/1/2017 | USA | 2000 |
| KY348852 | 15412 | 3/1/2017 | USA | 2000 |
| KU131569 | 15411 | 12/31/2016 | USA | 1999 |
| KT257991 | 15020 | 7/11/2016 | USA | 2014 |
| KT257990 | 15069 | 7/11/2016 | USA | 2014 |
| KT257986 | 14917 | 7/11/2016 | USA | 2014 |
| KT257984 | 14917 | 7/11/2016 | USA | 2014 |
| KT257982 | 15009 | 7/11/2016 | USA | 2014 |
| KT257981 | 15009 | 7/11/2016 | USA | 2014 |
| KT257980 | 15015 | 7/11/2016 | USA | 2014 |
| KT257978 | 14872 | 7/11/2016 | USA | 2014 |
| KT257976 | 15127 | 7/11/2016 | USA | 2014 |
| KT257975 | 15025 | 7/11/2016 | USA | 2014 |
| KT257974 | 14965 | 7/11/2016 | USA | 2014 |
| KT257973 | 15002 | 7/11/2016 | USA | 2014 |
| KT257972 | 15018 | 7/11/2016 | USA | 2014 |

| | | | | |
|---|---|---|---|---|
| KT257971 | 15075 | 7/11/2016 | USA | 2014 |
| KT257970 | 15018 | 7/11/2016 | USA | 2014 |
| KT257965 | 14967 | 7/11/2016 | USA | 2014 |
| KT257964 | 15019 | 7/11/2016 | USA | 2014 |
| KT257963 | 15146 | 7/11/2016 | USA | 2014 |
| KT257962 | 14967 | 7/11/2016 | USA | 2014 |
| KT257959 | 15122 | 7/11/2016 | USA | 2014 |
| KT207837 | 15071 | 1/24/2016 | USA | 5-Nov-12 |
| KF724413 | 15335 | 4/23/2015 | USA | 11-Mar-13 |
| KF724412 | 14916 | 4/23/2015 | USA | 8-Mar-13 |
| KF724411 | 14943 | 4/23/2015 | USA | 13-Mar-13 |
| KF724410 | 14868 | 4/23/2015 | USA | 13-Mar-13 |
| KF724409 | 14944 | 4/23/2015 | USA | 5-Mar-13 |
| KF724408 | 14945 | 4/23/2015 | USA | 5-Mar-13 |
| KF724407 | 15325 | 4/23/2015 | USA | 26-Feb-13 |
| KF724406 | 15155 | 4/23/2015 | USA | 19-Dec-11 |
| KF724404 | 15151 | 4/23/2015 | USA | 15-May-12 |

| | | | | |
|---|---|---|---|---|
| KF724403 | 15363 | 4/23/2015 | USA | 21-May-12 |
| KF724402 | 14787 | 4/23/2015 | USA | 12-Mar-12 |
| KF724401 | 14785 | 4/23/2015 | USA | 9-Feb-12 |
| KF724400 | 14755 | 4/23/2015 | USA | 9-Feb-12 |
| KF724399 | 15177 | 4/23/2015 | USA | 4-Jan-12 |
| KF724398 | 14778 | 4/23/2015 | USA | 6-Dec-11 |
| KF724397 | 14813 | 4/23/2015 | USA | 21-Dec-11 |
| KF632717 | 14918 | 4/23/2015 | USA | 26-Feb-12 |
| KP283416 | 14991 | 4/22/2015 | USA | 2012 |
| KP283415 | 15004 | 4/22/2015 | USA | 2012 |
| KP283414 | 15004 | 4/22/2015 | USA | 2012 |
| KP283413 | 14998 | 4/22/2015 | USA | 2012 |
| KP283412 | 15005 | 4/22/2015 | USA | 2012 |
| KP283411 | 15001 | 4/22/2015 | USA | 2012 |
| KP283410 | 15000 | 4/22/2015 | USA | 2012 |
| KP283409 | 15002 | 4/22/2015 | USA | 2012 |
| KP283408 | 15001 | 4/22/2015 | USA | 2012 |

| | | | | |
|---|---|---|---|---|
| KP283407 | 15002 | 4/22/2015 | USA | 2012 |
| KP283406 | 15003 | 4/22/2015 | USA | 2012 |
| KP283405 | 15001 | 4/22/2015 | USA | 2012 |
| KP283404 | 15003 | 4/22/2015 | USA | 2011 |
| KP283403 | 14999 | 4/22/2015 | USA | 2012 |
| KP283402 | 15002 | 4/22/2015 | USA | 2013 |
| KP283401 | 14998 | 4/22/2015 | USA | 2012 |
| KP283400 | 15001 | 4/22/2015 | USA | 2012 |
| KP283399 | 15002 | 4/22/2015 | USA | 2012 |
| JQ087873 | 15470 | 8/23/2012 | USA | 2010 |
| HQ699067 | 15389 | 2/15/2012 | USA | 2006 |
| MN175678 | 15433 | 12/22/2019 | USA | 2016 |
| MN175677 | 14915 | 12/22/2019 | USA | 2016 |
| MK837936 | 15412 | 11/19/2019 | USA | 1996 |
| MF663706 | 15063 | 10/3/2018 | USA | 2016 |
| KR709268 | 6148 | 10/25/2015 | USA | 2014 |
| KR709267 | 6117 | 10/25/2015 | USA | 2014 |

| | | | | |
|---|---|---|---|---|
| KR709266 | 6144 | 10/25/2015 | USA | 31-Dec-14 |
| KR709265 | 6144 | 10/25/2015 | USA | 31-Dec-14 |
| KR709264 | 6143 | 10/25/2015 | USA | 30-Dec-14 |
| KR709263 | 6128 | 10/25/2015 | USA | 22-Nov-14 |
| KR709262 | 6147 | 10/25/2015 | USA | 22-Nov-14 |
| KT258000 | 14971 | 7/11/2016 | USA: Colorado | 2014 |
| KT257999 | 14971 | 7/11/2016 | USA: Colorado | 2014 |
| KT257955 | 15017 | 7/11/2016 | USA: Colorado | 2014 |
| MF526965 | 15081 | 6/23/2018 | USA: Indiana | 1-Jun-17 |
| MF526964 | 15079 | 6/23/2018 | USA: Indiana | 1-Feb-17 |
| MF326992 | 15111 | 12/2/2017 | USA: Indiana | 2014 |
| MK820651 | 15395 | 11/13/2019 | USA: Indiana | 1995 |
| MF327001 | 15110 | 12/2/2017 | USA: Iowa | 2015 |
| MF327000 | 15062 | 12/2/2017 | USA: Iowa | 2015 |
| MF326997 | 15110 | 12/2/2017 | USA: Iowa | 2015 |
| MF326996 | 15062 | 12/2/2017 | USA: Iowa | 2015 |
| MF326995 | 15110 | 12/2/2017 | USA: Iowa | 2014 |

| | | | | |
|---|---|---|---|---|
| MF326989 | 15408 | 12/2/2017 | USA: Iowa | 2014 |
| MF326988 | 15017 | 12/2/2017 | USA: Iowa | 2013 |
| MF326987 | 15107 | 12/2/2017 | USA: Iowa | 2015 |
| MF326986 | 15110 | 12/2/2017 | USA: Iowa | 2015 |
| MF326985 | 15110 | 12/2/2017 | USA: Iowa | 2014 |
| KT258009 | 14971 | 7/11/2016 | USA: Iowa | 2014 |
| KT258004 | 15002 | 7/11/2016 | USA: Iowa | 2014 |
| KT258003 | 15002 | 7/11/2016 | USA: Iowa | 2014 |
| KT258002 | 15018 | 7/11/2016 | USA: Iowa | 2014 |
| KT258001 | 15002 | 7/11/2016 | USA: Iowa | 2014 |
| KT257993 | 14983 | 7/11/2016 | USA: Iowa | 2014 |
| KT257987 | 14986 | 7/11/2016 | USA: Iowa | 2014 |
| KT257983 | 14958 | 7/11/2016 | USA: Iowa | 2014 |
| KT257977 | 15019 | 7/11/2016 | USA: Iowa | 2014 |
| KT257968 | 15122 | 7/11/2016 | USA: Iowa | 2014 |
| KT257967 | 15132 | 7/11/2016 | USA: Iowa | 2014 |
| KT257947 | 15410 | 7/11/2016 | USA: Iowa | 2014 |

| | | | | |
|---|---|---|---|---|
| KT257946 | 15411 | 7/11/2016 | USA: Iowa | 2014 |
| KT257945 | 15409 | 7/11/2016 | USA: Iowa | 2014 |
| KT257944 | 15412 | 7/11/2016 | USA: Iowa | 2014 |
| KT257958 | 14918 | 7/11/2016 | USA: Kansas | 2014 |
| KT257956 | 15013 | 7/11/2016 | USA: Kansas | 2014 |
| KT257954 | 15015 | 7/11/2016 | USA: Kansas | 2014 |
| KT257953 | 15412 | 7/11/2016 | USA: Minnesota | 2014 |
| KT257952 | 15396 | 7/11/2016 | USA: Minnesota | 2014 |
| KT257950 | 15412 | 7/11/2016 | USA: Minnesota | 2014 |
| KT257948 | 15390 | 7/11/2016 | USA: Minnesota | 2014 |
| KT257992 | 15001 | 7/11/2016 | USA: Missouri | 2014 |
| KX192119 | 15097 | 6/7/2016 | USA: Nebraska | 2016 |
| KX192117 | 15097 | 6/7/2016 | USA: Nebraska | 2016 |
| KX192116 | 15097 | 6/7/2016 | USA: Nebraska | 2016 |
| KX192115 | 15098 | 6/7/2016 | USA: Nebraska | 2016 |
| KX192114 | 15097 | 6/7/2016 | USA: Nebraska | 2016 |
| KX192113 | 15101 | 6/7/2016 | USA: Nebraska | 2016 |

| | | | | |
|---|---|---|---|---|
| KX192112 | 15097 | 6/7/2016 | USA: Nebraska | 2016 |
| KX192118 | 15098 | 6/7/2016 | USA: Nebraska | 2016 |
| KT258007 | 15002 | 7/11/2016 | USA: Nebraska | 2014 |
| KT257998 | 14981 | 7/11/2016 | USA: Nebraska | 2014 |
| KT257997 | 15013 | 7/11/2016 | USA: Nebraska | 2014 |
| KT257996 | 14890 | 7/11/2016 | USA: Nebraska | 2014 |
| KT257995 | 14981 | 7/11/2016 | USA: Nebraska | 2014 |
| KT257994 | 14981 | 7/11/2016 | USA: Nebraska | 2014 |
| MF326999 | 15014 | 12/2/2017 | USA: North Carolina | 2015 |
| MF326998 | 15071 | 12/2/2017 | USA: North Carolina | 2015 |
| MF326991 | 15110 | 12/2/2017 | USA: North Carolina | 2014 |
| MF326990 | 15110 | 12/2/2017 | USA: North Carolina | 2014 |
| KT258006 | 15012 | 7/11/2016 | USA: North Carolina | 2014 |
| KT257966 | 15070 | 7/11/2016 | USA: North Carolina | 2014 |
| MK860181 | 15110 | 11/20/2019 | USA: North Carolina | 2015 |
| KR534894 | 15110 | 12/8/2015 | USA: Ohio | 2-Jan-15 |
| KR534893 | 15009 | 12/8/2015 | USA: Ohio | 5-Dec-13 |

| | | | | |
|---|---|---|---|---|
| MF326994 | 15110 | 12/2/2017 | USA: Ohio | 2014 |
| MF326993 | 15019 | 12/2/2017 | USA: Ohio | 2014 |
| KU842720 | 15276 | 3/8/2016 | Vietnam | 22-Feb-10 |
| JX512910 | 15320 | 3/23/2018 | Vietnam | 2007 |
| MG251835 | 15100 | 9/12/2018 | Vietnam | 1-Jul-16 |
| MG251834 | 15100 | 9/12/2018 | Vietnam | 1-Jul-16 |
| MG251833 | 15100 | 9/12/2018 | Vietnam | 1-Jul-16 |
| LC569874 | 15230 | 7/23/2020 | Vietnam | 2010 |

**Supplementary Table 2 .** Scientific productivity, measured as the total number of articles using PRRSV whole genome and single genes per country.

| Country | ORF5 | Whole genome |
|---|---|---|
| Argentina | 0 | 1 |
| Australia | 2 | 4 |
| Austria | 3 | 4 |
| Belarus | 1 | 0 |
| Belgium | 4 | 9 |

| | | |
|---|---|---|
| Brazil | 1 | 0 |
| Bulgaria | 0 | 1 |
| Cameroon | 2 | 0 |
| Canada | 12 | 16 |
| Chile | 6 | 0 |
| China | 141 | 295 |
| Croatia | 0 | 1 |
| Czech Republic | 3 | 1 |
| Denmark | 8 | 11 |
| Egypt | 1 | 0 |
| France | 7 | 14 |
| Georgia | 1 | 0 |
| Germany | 5 | 5 |
| Greece | 3 | 0 |
| Hungary | 4 | 15 |
| India | 6 | 0 |
| Ireland | 1 | 2 |
| Italy | 11 | 16 |
| Japan | 10 | 9 |

| | | |
|---|---|---|
| Kuwait | 1 | 1 |
| Lithuania | 2 | 1 |
| Malaysia | 5 | 0 |
| Mexico | 5 | 3 |
| Myanmar | 0 | 2 |
| Netherlands | 4 | 0 |
| New Zealand | 1 | 1 |
| Norway | 0 | 3 |
| Peru | 2 | 0 |
| Poland | 1 | 12 |
| Romania | 2 | 2 |
| Serbia | 0 | 3 |
| Slovakia | 1 | 2 |
| Slovenia | 0 | 1 |
| South Africa | 1 | 1 |
| South Korea | 50 | 55 |
| Spain | 13 | 6 |
| Sweden | 3 | 2 |
| Switzerland | 0 | 4 |

| | | |
|---|---|---|
| Thailand | 17 | 13 |
| Uk | 18 | 12 |
| Uruguay | 3 | 0 |
| Usa | 88 | 89 |

1. **Supplementary Table 3.** Phylodynamic results describing the dispersion statistics of PRRSV, using whole genome sequences (WGS), and ORF5 sequences.

| Variable | WGS | | ORF5 | |
|---|---|---|---|---|
| | Mean (km/year) | 95% HPD (km/year) | Mean (km/year) | 95% HPD (km/year) |
| Median value of mean branch dispersal velocity | 1956.4 | 1646.0, 2666.2 | 2948.7 | 1834.1, 6525.0 |
| Median value of weighted branch dispersal velocity | 359.1583 | 300.6, 477.7 | 503.2 | 231.9, 1276.9 |
| Median value of original diffusion coefficient | 1138956 | 914426.3, 1517280.0 | 442836.7 | 85913.3, 2433036.1 |
| Median value of weighted diffusion coefficient | 505466.1 | 441933.7, 749903.2 | 497245.9 | [185468.1, 1833533.2 |
